# Conformational analysis, molecular structure, spectroscopic, NBO, reactivity descriptors, wavefunction and molecular docking investigations of 5,6-dimethoxy-1-indanone: A potential anti Alzheimer's agent

**DOI:** 10.1016/j.heliyon.2022.e08821

**Published:** 2022-01-23

**Authors:** S. Sebastian, S. Sylvestre, N. Sundaraganesan, B. Karthikeyan, S. Silvan

**Affiliations:** a P.G & Research Department of Physics, St.Joseph's College of Arts & Science (Autonomous), Cuddalore, 607001, Tamilnadu, India; b Department of Chemistry, School of Mathematics and Natural Sciences, Mukuba University, Kitwe, 20382, Zambia; c Department of Physics (Engg.), Annamalai University, Annamalai Nagar, 608 002, India; d Department of Chemistry, Annamalai University, Annamalai Nagar, 608 002, India; e P.G & Research Department of Biochemistry, St.Joseph's College of Arts & Science (Autonomous), Cuddalore, 607001, Tamilnadu, India

**Keywords:** 5,6-dimethoxy-1-indanone, Vibrational spectra, Wavefuntion, Molecular docking, Alzheimer's disease

## Abstract

The objective of the present study is focused to elucidate the structure of potential anti-Alzheimer's compound 5,6-Dimethoxy-1-indanone (5,6-DMI) and study its binding interaction towards the active site by molecular docking studies. The structural and various spectroscopic tools are used to understand the various interaction behaviors of the title compound. The theoretical calculation of 5,6-DMI molecule is computed by Gaussian 09W software with Density functional B3LYP and CAM-B3LYP method utilizing 6-311G(d,p) as basis set. The Natural Bond Orbital (NBO) analysis has been performed to find all possible transition was correlate with electronic transition. The Non covalent interaction of 5,6-DMI molecule was examined by adopt Reduced Density Gradient (RDG) analysis and colour filled ELF diagram. Molecular docking results suggest that 5,6-DMI may exhibit inhibitory activity against apoE protein and may act as potential against Alzheimer's disease.

## 1 Introduction

Alzheimer's disease (AD) is a social threat and progressive neurodegenerative disorder and one of the most universal causes of mental weakening in the early age of human being. Recent research efforts are to study the drug development, determination of molecular, biochemical and cellular mechanisms of AD. Several hypotheses proposed to elucidate the pathogenic characterization of AD including β-amyloid deposition, tauhyperphosphorylation, acetylcholine deficiency, inflammation, and oxidative stress. The Acetyl Cholinesterase (AChE) inhibitors are being major and large amount developed class of drugs approved for AD therapy have been approved by Food and Administration (FDA) and European Medicines Evaluation Agency (EMEA) example such as donepezil, rivastigime and galanthamine for symptomatic treatment of behavioural and psychiatric symptoms of AD [1]. The indanone derivatives play an important part in the discovering of novel structural moiety for the action of AChE inhibitors [2]. The indanone derivative are seems to be interesting chemical used to synthesis some important biomedical applications oriented compound such as anticonvulsants [3] anticholinergics [4] and aromatic retinoids [5].

The conformational stability and vibrational spectral studies of 2-bromo-1-indanol based on DFT has been investigated by Balchandran et al. [6], based on quantum chemical calculation. For 5-fluro-1-indanone molecule, the various spectral measurements have been recorded at different temperatures and states of aggregation were also carried out by F.Gomez et.al [7]. by assume C_s_ as point group. T.P.Ruiz et al. [8] studied the structure of 1-indanone by XRD at 120 ​K and calculated its vibrational frequencies and theoretical calculations by DFT techniques. Intermolecular forces on crystal structure of 5-chloro-1-indanone are reported by T.P.Ruiz [9] et al. Several noval derivatives of 5,6-DMI was synthesized based on Schiff's are found in literature by V.M.Patel et. al. [10], they also found all the derivative shows potential antimicrobial agents. M.Tureik et. al., [11], reported the comprehensive methods of preparation of 1-indanones in research article and patent for a decade of years. Up to our knowledge, only the XRD study of 5,6-DMI was reported by Shoja et al. [12] so far. In our present study we are focused on determining the molecular structure, a detailed vibrational (FTIR and FT-Raman), NMR and UV-Vis spectroscopic studies of 5,6-DMI theoretically. NBO population analysis was carried out on 5,6-DMI to understand the electronic properties. The TD-DFT calculations were performed for 5,6-DMI molecule along with HOMO-LUMO energy used to find various reactive descriptors. Wavefunction analysis on the molecule used to determine the various interactions in the molecular system. Molecular docking studies were also performed for 5,6-DMI for understanding the binding interaction of the compound.

## 2 Materials and methods

### 2.1 Experimental details

**Figure 1:**
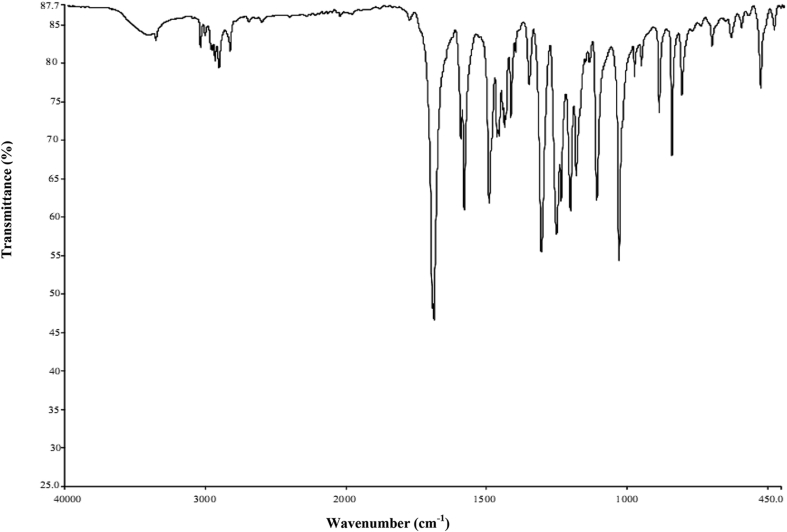
FT-IR spectrum of 5,6-DMI.

**Figure 2:**
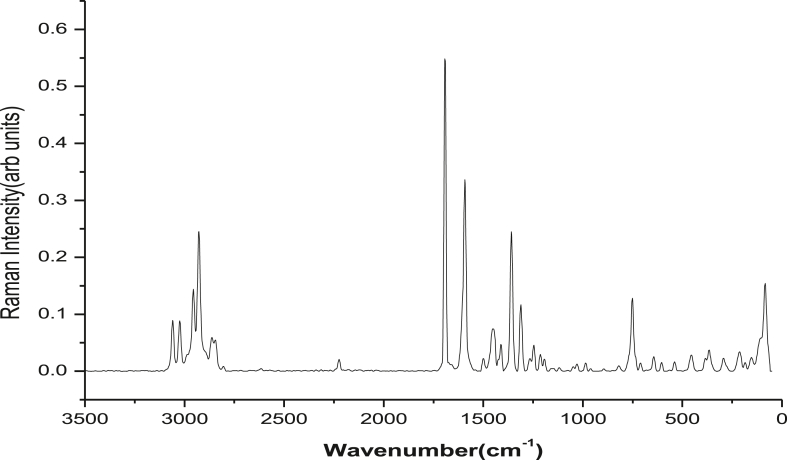
FT- Raman spectrum of 5,6-DMI.

**Figure 3:**
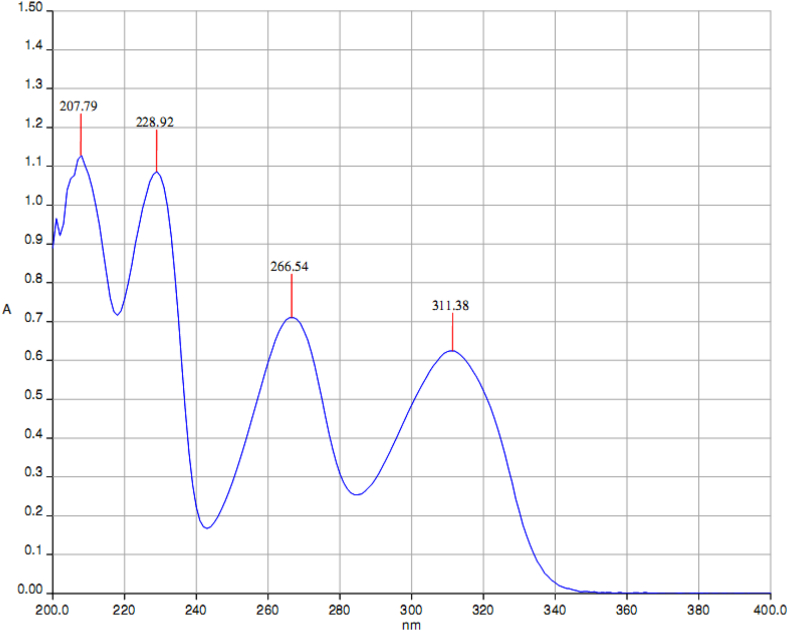
Experimental UV-Vis spectrum of 5,6-DMI.

The compound 5,6-dimethoxindanone (5,6-DMI) were purchased from Sigma Aldrich chemicals with assay of 98% and used without further purification. The FT-IR spectrum of title compound were recorded between the region 4000–400 cm^−1^ using IFS 66 ​V spectrophotometer as shown in Figure 1. The spectrum of FT-Raman was recorded using Nd: YAG laser (1064 nm) line as a excitation wavelength between the region 3500-50 cm^−1^ using Thermo Electron Corporation model Nexus 670 spectrophotometer as shown in Figure 2. The proton NMR spectrum were recorded for the title compound at 300 MHz on AV300 NMR spectrometer at room temperature and the ^13^C NMR also recorded on the same instrument as shown in Figure S1 (a) and (b). TMS proton spectrum has the following experimental parameters. Number of scan 16: spectral width 6172.84 Hz; acquisition time 2.65s. ^1^H NMR spectrum has the following experimental parameters: number of scan 127; spectral width 17985.61 Hz acquisition time 1.82s. The SHIMADZU UV-1650 PC instrument are used to record UV-Vis spectra between the region 200–400 nm using ethanol as a solvent phase as shown in Figure 3.

### 2.2 Computational details

The Density Functional Theory (DFT) plays an efficient theory for solving electronic structure of molecules. The CAM-B3LYP/6-311G(d,p) and B3LYP/6-311G (d,p) method augmented by ‘d’ polarization function and ‘p’ diffuse function for heavy and hydrogen atoms to describe the polar bonds of molecules [13, 14]. The entire calculations are carried out by Gaussian 09W [15] program using default convergence criteria [16] implemented in the software. In order to understand molecular behavior, various conformers of 5,6-DMI has to be found. The assignment for computed vibrational wavenumber is carried out by finding Total Energy Distribution using VEDA program package [17].

The Natural Bond Orbital (NBO) analysis have been done by using NBO 5.0 [18] program which is integrated in Gaussian 09W [15] package at CAM-B3LYP/6-311G(d,p) method. The ^1^H and ^13^C NMR isotropic chemical shifts for 5,6-DMI were calculated with GIAO method [19, 20]. The TD-DFT calculation is used to compute electronic parameters. In TD-DFT calculation solvent effect was included using IEF-PCM model author by Tomasi and Co-worker [21, 22]. The Gauss Sum 3.0 program [23] is used to draw the Density of State (DOS) spectra for gas and ethanol phase. Multiwfn [24] and VMD 9.1 Program [25] software is used to draw Reduced Density Gradient (RDG) and Electrostatic Potential Surface (EPS) maps.

### 2.3 Predication of Raman Intensity

The theoretical Raman spectrum was plotted by using the Eq. (1) [26, 27].

(1) $$I_{i}^{R}=C{\left(v_{o}-v_{i}\right)}^{4}v_{i}^{-1}B_{i}^{-1}S_{i}$$

where B_i_ is temperature factor that determine the intensity contribution of excited states to each vibration, Boltzmann appropriation can be found from Eq. (2),

(2) $$B_{i}=1-\exp\left(-\frac{hv_{i}C}{kT}\right)$$

In $I_{i}^{R}$ equation, *v*_*o*_ is frequency of laser beam (1064 nm of Nd: YAG laser), *v*_*i*_ denote the normal mode of frequency (cm^−1^), S_i_ is Raman scattering activity of the normal mode Q_i_. In above equations C, T, h and K are speed of light, temperature (in Kelvin), Planck and Boltzmann constants.

## 3 Results and discussions

### 3.1 Molecular geometry

The title compound 5,6-DMI is monoclinic system having space group *P*_*21*_*/c,* with Z = 4, and cell parameters a = 8.173(2)Ǻ, b = 6.003(1) Ǻ, c = 20.034 (4) Ǻ, β = 96.75(3)°, V = 976.1(5) Ǻ was reported by Shoja et al. [12]. The 5,6-DMI is aromatic heterocyclic molecule and becomes a great concern due to two methoxy group present in the ring system. The starting parameters for computations were constructed by considering the crystallographic data reported by Shoja et al. [12].

**Figure 4:**
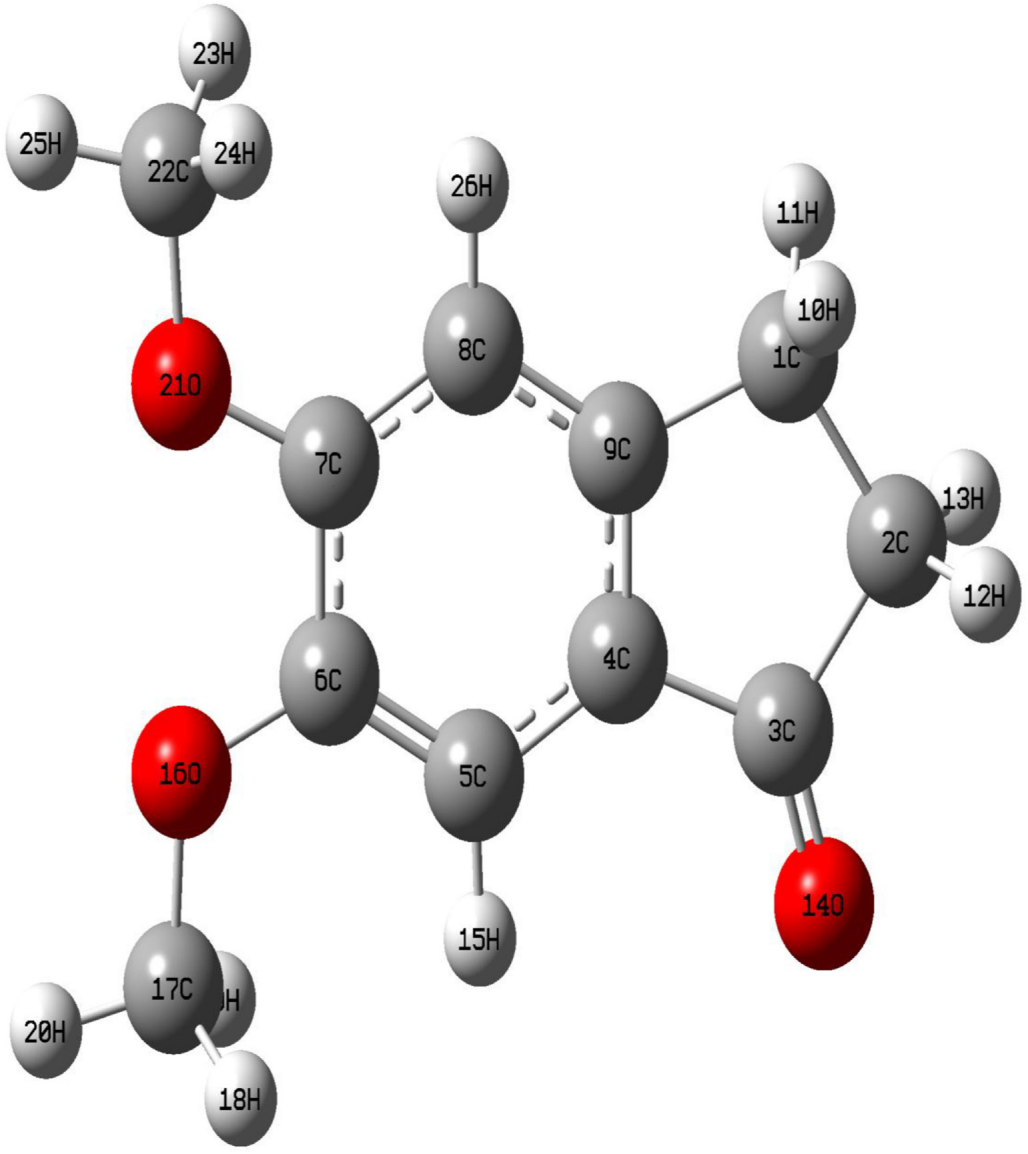
Molecular structure and atom numbering scheme adopted in this study for 5,6-DMI.

The Figure S2 shows the different possible conformer along with energy values in kcalmol^−1^ by CAM-B3LYP/6-311G(d,p) method, among the conformers, we identify the most stable conformer is Conformer 1 (relative energy of other conformer is determined by compare the most stable conformer energy with other conformers energy) by CAM-B3LYP/6-311G(d,p) method as shown in Table S1, the least stable conformer is found for conformer 5 with relative stable energy of 2.79405 kcalmol^-1^, for conformer 2 is 1.61738 kcalmol^-1^, conformer 3 and 4 have the relative stable energy of 1.41567 and 1.61733 kcalmol^-1^ as shown in Figure S2. The study of different conformations of 5,6-DMI molecule based on its energy difference, the Conformer 1 is the most stable conformer which implies that the orientation of OCH_3_ is orient opposite plane will provide the least stable conformer is well coincide with already reported molecular structure 5,6-DMI by Shoja et al. [12] and the results are once again confirmed by Potential Energy Surface scan study in the following discussion. The single crystal XRD and optimized molecular geometry of 5,6-DMI was tabulated in Table 1 for C1 conformer by both the method and reported XRD by Shoja et al. [12] for the title molecule are shown in the counterpart. The most stable conformer C1 conformer is are shown in Figure 4.

**Table 1:** Geometrical parameters optimized in 5,6-DMI [bond length (Å), bond angle (◦) and dihedral angle (◦)] by DFT method.

Parameters	B3LYP/6-311G(d,p)	CAM-B3LYP/6-311G(d,p)	aXRD	Parameters	B3LYP/6-311G(d,p)	CAM-B3LYP/6-311G(d,p)	aXRD
**Bond length (Ǻ)**				**Bond angle (**^**o**^**)**			
C1–C2	1.546	1.540	1.541(5)	C5–C4–C9	121.8	122.0	122.2(3)
C1–C9	1.516	1.511	1.503(4)	C4–C5–C6	118.9	118.7	118.1(3)
C2–C3	1.539	1.529	1.521(4)	C5–C6–C7	119.5	119.6	120.0(3)
C3–C4	1.473	1.471	1.468(4)	C5–C6–O16	125.5	125.4	125.7(2)
C3–O14	1.213	1.206	1.211(4)	C7–C6–O16	114.9	114.9	114.3(2)
C4–C5	1.403	1.398	1.401(4)	C6–C7–C8	120.5	120.6	121.3(3)
C4–C9	1.386	1.376	1.385(4)	C6–C7–O21	114.7	114.6	113.7(2)
C5–C6	1.383	1.375	1.376(4)	C8–C7–O21	124.7	124.6	125.0(3)
C6–C7	1.431	1.425	1.425(4)	C7–C8–C9	119.2	119.0	118.5(3)
C6–O16	1.358	1.352	1.361(4)	C1–C9–C4	111.7	111.8	112.1(3)
C7–C8	1.393	1.384	1.380(4)	C1–C9–C8	128.3	128.3	127.9(3)
C7–O21	1.353	1.346	1.364(4)	C4–C9–C8	119.8	119.8	120.0(3)
C8–C9	1.397	1.394	1.397(4)	C6–O16–C17	117.6	117.5	116.7(2)
C16–C17	1.421	1.412	1.424(4)	C7–O21–C22	118.5	118.4	117.3(2)
O21–C22	1.421	1.412	1.424(4)	**Dihedral angle (**^**o**^**)**			
**Bond angle (**^**o**^**)**				C9–C1–C2–C3	0.0	0.0	2.8(3)
C2–C1–C9	104.3	104.2	104.3(2)	C2–C1–C9–C4	0.0	0.0	-3.0(3)
C1–C2–C3	106.2	106.2	106.1(3)	C2–C1–C9–C8	180.0	0.0	-3.0(3)
C2–C3–C4	106.9	107.0	107.7(2)	C1–C2–C3–C4	0.0	179.9	-1.7(3)
C2–C3–O14	125.7	125.8	125.5(3)	C2–C3–C4–C9	0.0	0.0	-0.1(3)
C4–C3–O14	127.2	127.1	126.7(3)	C3–C4–C9–C1	0.0	0.0	2.0(3)
C3–C4–C5	127.4	127.3	128.1(3)	C8–C7–O21–C22	0.0	0.0	-2.4(4)
C3–C4–C9	110.6	110.5	109.7(2)	C7–O16–C6–C5	0.0	0.0	-10.9(4)

a Taken from ref [12].

In Indanone moiety, methoxy group attached to C6 atom is oriented out-of-plane to the aromatic ring [12] with torsion angle [C17–O16–C6–C5 = -10.9°(4)], while the computed results shows the O16CH_3_ is not twisted. For the ring angle concerned C3–C4–C9–C1 = 0.00° deviate ∼2° with that of XRD value, this may due to the single molecular are consider for computation in gas phase while real system is in condensed phase.

The substitution of hydrogen atom by methoxy group in 5,6-DMI play a crucial role in the interatomic distances, particularly for ring C–C bond distance compared to computed C=C bond distances. The methoxy oxygen atoms i.e O16 and O21 have lone pair electrons which leads to decrease the bond length between C6–O16 = 1.361(4) Ǻ and C7–O21 = 1.364(4) Ǻ shows a deviation of about 0.003 and 0.011Ǻ when compared to computed parameters by B3LYP/6-311G(d,p) method [C6–O16 = 1.358 Ǻ and C7–O21 = 1.353 Ǻ] and 1.352 Ǻ and 1.346 Ǻ by CAM-B3LYP/6-311G(d,p) method as shown in Table 1, this can be again proved by the calculated bond angles of the aromatic i.e. the symmetry of the rings is slightly distorted from the normal angle of 120° at the ortho and meta positions of the aromatic ring [C5–C6–C7 = 119.5°, C6–C7–C8 = 120.5° and C5–C4–C9 = 121.8°, C4–C9–C8 = 119.8°], that may leads to the cyclopentanone moiety to the out-of-plane. The C=O (C3 = O14) bond length is equal to 1.211 (4) Ǻ value is found to be correlate with standard literature data for C=O bond length [28, 29] as well as computed bond length of 1.213 Ǻ. All C–C bond distances of six member ring are falls in the range 1.383–1.431 Ǻ, while the C–C bond distance of the five membered ring is lies between 1.473-1.546 Ǻ. The C–H bond distance of the ethylene group is lies between 1.093 - 1.095 Ǻ.

### 3.2 NBO charge analysis

NBO charge analysis were carried for 5,6-DMI molecule predicated by B3LYP/6-311G(d,p) and CAM-B3LYP/6-311G(d,p) method are tabulated in Table S2 and the same is illustrated in Figure S3 (a) and (b). The study serves as key to identify the formation of molecular system because charges affect structure of a molecule, dipole moment and other similar properties. As we noted in Table S2, in 5,6-DMI the five membered ring have more electron when compared to phenyl ring and also methoxy group attached in plane of hydrogen act as electron donating group, it exhibit –I (negative inductive effect) C7 (0.3368e) and C6 (0.2964e) is more positive charge when compare to five membered ring C7 (−0.1543e) and C6 (−0.2003e) by B3LYP/6-311G(d,p) method due to OCH_3_ (high electronegative) group. By comparing these charge difference between Indanone and 5,6-DMI, the high negative charge at C6 and C7 in 5,6-DMI and large positive charge at C6 and C7 in indanone reveals the high electron attracting nature of OCH_3_ (methoxy) group leads to possible delocalization of electron towards these group. In case of five membered ring both molecules does not show not much change in charge distribution as evident from Table S2. The natural atomic charge revealed that O14, O16 and O21 atoms are more electronegativity at this bond interacts with receptor by hydrogen bond during molecular docking. The NBO charge of indanone and 5,6-DMI are shown in Figure S3 (a) and (b) for comparison.

### 3.3 Potential energy surface scan

**Figure 5:**
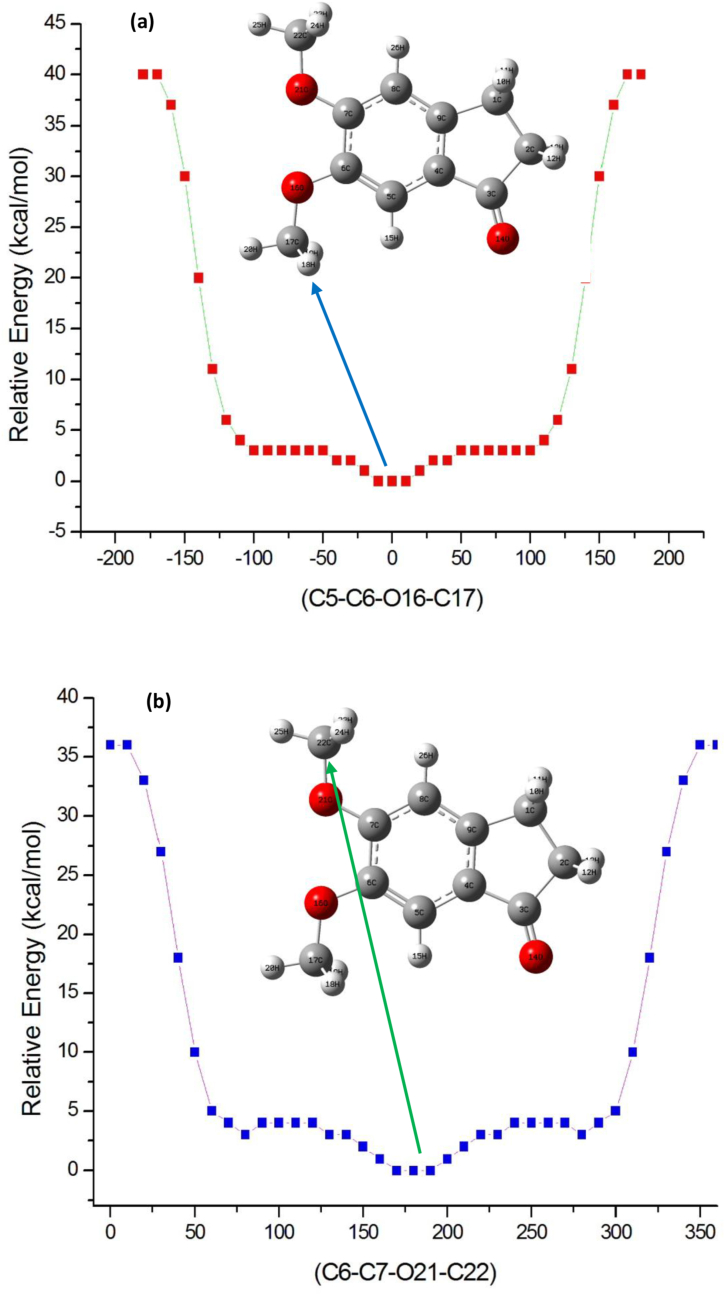
PES scan for dihedral angle vs relative energy for dihedral angle C5–C6–O16–C17 (a) and C6–C7–O21–C22 (b) at CAM-B3LYP/6-311G (d,p) method for 5,6-DMI.

The Potential Energy Surface (PES) scan for OCH_3_ group (O16C17H_3_ and O21C22H_3_) is carried out for 5,6-DMI molecule by CAM-B3LYP/6-311G(d,p) method. As we seen form the Figure 5 (a) and (b) only the two methoxy group (O16C17H_3_ and O21C22H_3_) is rotatable coordinate found in the 5,6-DMI molecule. While doing calculations all internal geometrical parameters are relaxed and the dihedral angle C5–C6–O16–C17 is varied from -180^o^ to 180^o^ and for C6–C7–O21–C22 dihedral angle is varied from 0^o^ to 360^o^ rotation around the bond. From Figure 5 (a) and (b) we found that for O16C17H_3_ have one global minimum energy is noticed at 0°, with energy value of 0.0589 kcal/mol and for O21C22H_3_ also one global minimum energy will be obtained as 0.0825 kcal/mol at 180°, the PES results shows that both OCH_3_ group are lies in the opposite plane with same energy, the obtained result correlate with conformer C1 as well as reported XRD structure of 5,6-DMI. by Shoja et al. [12].

### 3.4 NBO analysis

The DFT level calculation is one of the promising method used to study NBO analysis. The natural orbital transition takes place between indanone ring and methoxy group. The hyperconjugative is the interaction of the electron in σ bond with neighboring antibonding π orbital's. This interaction is possible for 5,6-DMI molecule because OCH_3_ group is directly attached to the indanone ring through n→π∗.

The NBO analysis shows that larger the E^(2)^ values, it clearly indicated that the molecule having more intensive interaction happen between electron donors and electron acceptors in the molecule. The Electron Density (ED) of double and single bond conjugation for 5,6-DMI (1.7–1.9e) clearly denote very strong delocalization happened in molecule. It is observed that a strong stabilizing intramolecular hyperconjugative interaction results of σ and π electron to C–C bond and anti bond in the indanone ring leads to stabilization energy in so me region of the ring system from Table S3. For example the σ [(C5–C6)] distribute to σ∗ [(C4–C5) and (C6–C7)] lead to stabilization energy of ∼5 kJ/mol. This further enhanced conjugated with antibonding orbital of π∗(C4–C9, C7–C8) leads to strong delocalization of 24.85 and 25.95 as shown in Table S3.

In 5,6-DMI, it is well observed a strong stabilizing intermolecular hyperconjugative interaction of C2–C3 from LP(2)O14 → σ∗(C2–C3) and σ∗(C4–C5) which ED (∼0.077e) that weakness the respective bonds of C2–C3 and C4–C5 leading to stabilization energy of 26.95 and 22.81 kJ/mol and also strong intermolecular hyperconjugative interaction of C5–C6 from LP(2)O16→ σ∗(C5–C6) and π∗(C5–C6) which ED (0.023 and 0.329e) that weakness the respective bonds of C5–C6 leading to stabilization energy of 7.72 and 37.68 kJ/mol.

The obtained NBO results describe the bonding nature of molecular system for example LP2(O14) occupy higher orbital energy (−0.30968a.u) with major p-character (99.90%) with low occupation number (1.87803) and other LP1(O14) occupy low energy orbital (−0.72465a.u) having p-character (45.11 %) and high occupation number (1.97673) as shown in Table 2. The LP2(O16) have higher orbital energy (−0.37106a.u) with significant p-character 99.95% with low occupation number (1.84640), another lower energy orbital (−0.60185a.u) with p-character 62.77% and high occupation number (1.96317). The hybrid orbital LP2 (O21) also shows the similar results as shown in Table 2. The NBO analysis also indicate pure p-type lone pair orbitals participate in electron contribution of LP1 (O14) → σ ∗(C2–C3), LP2(O16) → π∗(C5–C6), LP2 (O21) → π∗(C7–C8) interactions in the molecule.

**Table 2:** NBO result showing the formation of Lewis and non-Lewis orbitals for 5,6-DMI by CAM-B3LYP/6-311G(d,p) method.

Bond (A-B)	ED/Energy (a.u)	EDA%	EDB%	NBO	S%	P%
σ (C1–C2)	1.98114 -0.66518	50.40	49.60	0.7099(sp^2.68^)C+ 0.7043 (sp^2.56^)C	27.14 28.11	72.82 71.85
σ∗ (C1–C2)	0.00740 0.0074	49.60	50.40	(0.7043 sp^2.68^)C + (-0.7099 sp^2.56^)C	27.14 28.11	72.84 71.85
σ (C2–C3)	1.98204 -0.67684	52.27	47.73	0.7230sp(^2.74^)C + 0.6909sp(^2.07^)C	26.69 32.61	73.25 67.35
σ ∗(C2–C3)	0.06726 0.06726	47.73%	52.27	0.6909sp(^2.74^)C +-0.7230sp(^2.07^)C	26.69 32.61	73.25 67.35
σ (C3–O14)	1.99554 -1.15983	34.12	65.88	0.5841sp(^2.14^)C +0.8117sp(^1.22^)O	31.80 45.08	68.05 54.80
π (C3–O14)	1.98087 -1.15983	32.09	67.91	0.5665sp(^1.00^)C +0.8241sp(^1.00^)O	0.00 0.00	99.56 99.87
σ∗(C3–O14)	0.01129 0.01129	65.88	34.12	0.8117sp(^2.14^)C+ -0.5841sp(^1.22^)O	31.80 43.08	68.05 54.80
π∗(C3–O14)	0.15397 0.15397	67.91	32.09	0.8241sp(^1.00^)C+ -0.5665sp(^1.00^)O	99.56 -	0.44 99.87
σ (C3–C4)	1.97517 -0.71111	47.08	52.92	0.6861sp(^1.83^)C+ 0.7275 sp(^2.29^)C	35.32 30.38	64.63 69.57
σ (C5–C6)	1.97724 -0.31325	49.95	50.05	0.7067sp(^1.83^)C+ 0.7075sp(^1.50^)C	35.34 39.95	64.60 60.02
π (C5–C6)	1.72694 -0.31325	50.85	49.15	0.7131sp(^1.00^)C+ 0.7011sp(^1.00^)C	- -	99.94 99.94
σ ∗(C5–C6)	0.02417 0.02417	50.05	49.95	0.7075sp(^1.83^)C+ -0.7067sp(^1.50^)C	35.34 64.60	39.95 60.02
π∗(C5–C6)	0.31856 0.31856	49.15	50.85	0.7011sp(^1.00^)C+ -0.7131sp(^1.00^)C	- -	99.93 99.94
σ (C7–C8)	1.97638 -0.79028	49.95	50.05	0.7068sp(^1.52^)C+ 0.7074sp(^1.87^)C	39.73 34.78	60.24 65.17
π(C7–C8)	1.71145 -0.31464	45.35	54.65	0.6734sp(^1.00^)C+ 0.7393sp(^1.00^)C	-	99.94 99.95
σ∗ (C7–C8)	0.02514 0.02514	50.05	49.95	0.7074sp(^1.52^)C+ -0.7068sp(^1.87^)C	39.73 34.78	60.24 65.17
π∗(C7–C8)	0.35068 0.35068	54.65	45.35	0.7393sp(^1.00^)C+ -0.6734sp(^1.00^)C	- -	99.94 99.95
LP1 (O14)	1.97673 -0.72465			sp(^0.82^)	54.87	45.11
LP2 (O14)	1.87803 -0.30968			sp(^99.90^)	0.03	99.90
LP1 (O16)	1.96317 -0.60185			sp(^1.69^)	37.20	62.77
LP2 (O16)	1.84640 -0.37106			sp(^1.00^)	-	99.95
LP1 (O21)	1.96277 -0.60528			sp(^1.73^)	36.56	63.40
LP2 (O21)	1.832256 -0.37809			sp(^1.00^)	-	99.95

### 3.5 Vibrational spectral analysis

In order to get vibrational analysis of the 5,6-DMI molecule, we probed vibrational frequency analysis on the basis of predicting the wavenumber by B3LYP and CAM- B3LYP/6-311G(d,p) method and are collected in Table 3. According to theoretical calculations the symmetry element of 5,6-DMI are assumed to be C_1_ point group symmetry, all 72 vibrations are splits into 49 in-plane and 23 out-of-plane vibrations. The theoretically predicated IR and Raman spectra are shown in Figure S4 (a) and (b) & S5 (a) and (b). In our present study we used scaling factor of 0.967 and 0.9608 [30] for B3LYP/6-311G(d,p) and CAM-B3LYP/6-311G(d,p) method.

**Table 3:** Vibrational wavenumbers obtained for 5,6-DMI at B3LYP/6-311G(d,p) and CAM-B3LYP/6-311G(d,p) method [harmonic frequency (cm^−1^), IR _int_(Kmmol^−1^), Raman Intensity (Arb Units)].

Mode nos.	Experimental Wavenumber(cm^−1^)		Theoretical Wavenumber (cm^−1^)						TED (≥10%) Assignments
	FT-IR	FT-Raman	B3LYP scaled	IR _int_	Ram_Int_	CAM B3LYP scaled	IR _int_	Ram_Int_	
1			3101	6.49	32.62	3104	5.430	30.11	υ C5–H15(99)
2	3058vw	3059ms	3090	10.42	33.52	3096	7.504	29.26	υ C8–H26(99)
3			3035	22.90	71.74	3041	19.956	66.84	υ_asy_C22H_3_(92)
4	3024vw	3024ms	3033	21.78	62.05	3039	19.191	57.98	υ_asy_C22H_3_(92)
5	2983vw		2992	14.70	68.07	3005	11.001	59.62	υ_asy_C2H_2_(94)
6	2972vw		2963	41.40	31.46	2971	41.22	36.32	υ_asy_C22H_3_(94)
7			2961	35.98	30.91	2971	5.06	27.19	υ_asy_C17H_3_(98)
8	2953vw	2956ms	2956	19.72	149.78	2968	31.21	28.12	υ_sy_C2H_2_(98)
9			2954	11.43	43.17	2966	13.75	126.56	υ_asy_C1H_2_(94)
10	2925vw	2928s	2930	35.60	102.44	2943	28.95	81.66	υ_sy_C1H_2_(94)
11			2906	34.69	108.70	2912	30.13	96.94	υ_sy_C22H_3_(98)
12			2904	70.70	44.79	2910	61.05	40.96	υ_sy_C17H_3_(98)
13	1703vs	1692vs	1722	362.48	226.15	1762	371.26	194.30	υ C3 = O14(88)
14	1590vs	1592vs	1584	100.00	168.563	1616	65.18	115.72	υ C5–C9(11)+ υ C7–C8(21)+ υ C4–C5(15)+δC5C6C7(10)
15			1571	77.77	224.04	1602	104.91	272.15	υ C4–C9(37) + υC5-C9(22)+ δC5C6C7(13)
16	1501s	1500vw	1486	186.07	37.17	1506	251.89	42.76	δC6C5H15(12) + δC7C8H26(18)
17	1466m		1456	34.46	14.45	1461	50.23	3.35	δH18C17H19(30) + δH19C17H20(13)+)+ δH23C22H24(16)
18		1452m	1455	64.83	15.81	1454	60.42	13.49	δH18C17H19(25) + δH23C22H24(20)+ δH24C22H25(13)
19	1446w		1445	9.59	46.97	1451	27.94	49.89	δH18C17H19(19) + δH23C22H25(43)+ δH23C22H24(26)
20			1444	23.58	29.17	1443	11.24	44.56	γH24C22H25(40) + τC22-O21H24–H25(15)
21	1441w		1443	7.62	45.62	1443	5.89	55.10	δH10C17H11(79) + δH12C2H13(10)
22			1442	4.72	69.45	1442	8.53	43.95	δH18C17H20(43) + δH19C17H20(40)
23	1424m		1430	22.63	19.49	1433	7.60	20.91	δH18C17H20(10) + δH18C17H19(12) + δH19C17H20(14)+ δH23C22H25(11)+ δH23C22H24(14)+ δH24C22H25(15)
24	1407w	1411w	1409	2.06	84.11	1413	19.62	27.74	δH12C2H13(77)
25			1399	37.98	14.39	1406	4.30	53.48	υ C7–C8(10)
26	1359m		1346	32.26	254.70	1355	106.96	325.73	υ C5–C6(10) + υ C7–C8(28)
27	1316vs	1311vs	1290	376.52	98.24	1306	303.93	46.30	υ C3–C4(10) + υ C1–C9(10) + δH11C1C2(11)
28	1261vs		1252	200.47	9.48	1258	179.42	8.22	υ C8–C9(10) + υC4-C5(11) + υO21-C7(12) + δH11C1C2(14)
29	1245s	1246ms	1236	1.45	9.40	1246	81.61	17.34	γ H12C2H13(32)+ τ H13C2C1C9(18)
30	1212s	1213w	1227	107.87	33.28	1240	115.93	6.36	δH15C5C6(22) +δ H26C8C7(18)
31	1191s		1196	137.14	17.18	1207	83.74	36.38	υ O16–C6(14) + γ C17H18H19O16(15)
32		1195vw	1196	0.05	20.40	1199	0.01	19.84	γ H11C1C2(28)+ τ H10C1C9C4(26)+ γ C1C2C9H11(11)
33			1175	6.16	21.39	1183	1.22	25.28	γ C17H18H19O16(41)
34			1170	13.87	29.93	1175	10.79	21.73	γ C22H23H24O21(63)
35	1145w		1154	9.34	0.92	1160	17.04	1.345	δH15C5C6(20) +δ H26C8C2(15)
36			1133	0.97	6.56	1138	0.98	6.08	γ H18C17H20(14) + τ C17H19O16H20(82)
37			1132	0.42	14.15	1138	0.43	12.80	γ H23C22H25(14) + τ C22H24O21H25(82)
38	1118vs		1121	0.14	7.77	1128	0.23	7.23	γ H11C2C2(16)+ τ H10C1C9C4(16) + γ C1C2C9H11(21)
39			1097	118.98	9.57	1116	105.77	12.00	υ C4–C5(11) + υ C3–C4(11) + υ C1–C9(19)
40	1039vs	1035vw	1028	13.24	7.26	1055	5.54	9.33	υ C17–O16(20) + υ C22–O21(48)
41	984w	990vw	1021	89.79	7.06	1044	80.84	7.71	υ C17–O16(41) + υ C2–C3(13)
42			978	0.01	1.14	985	0.00	0.94	γ H12C2–C3 (12) + γ C1C2C9H11(36) + τ C3–C2C4C9 (20)
43	960w		965	4.15	24.79	977	3.93	20.58	υ C1–C2(55)
44	897s		944	1.16	4.84	960	0.14	9.56	υ C1–C2(25)
45			857	23.75	8.10	873	25.53	7.70	τ H15C5C4C3(77)
46	851s		831	22.88	7.13	844	24.15	7.45	τ H26C8C9C1(65) + τ H12C2C3O14(11)
47	815s	816vw	803	0.08	4.98	812	36.12	7.64	τ H26C8C9C1(16) + γ C1C2C9H11(10) + τ H12C2C2O14(31)
48			801	35.91	6.34	810	0.66	5.61	υ O16–C6(15) + υ C1–C9 (13) + υ C2–C3(10)
49	748w	752s	738	0.00	221.54	747	0.05	207.	υ C4–C9(18) + δC5C4C9(26)
50			712	0.00	0.81	724	0.00	0.67	τ C6C5C4C9(11) + γ O16C5C4C6(29) + γ O21C6C8C7(35)
51	709w		695	8.09	19.00	700	7.37	14.72	δC4C9C8(24) + δC3C4C9(10)
52	640w	644w	633	7.76	47.53	637	6.32	38.37	δC2C1C9(13) + δC3C4C9(25)
53	603w	605w	627	0.00	2.13	633	0.00	2.76	τ C6C5C4C9(13) + γ C3C5C9C4(28)
54	577vw		586	0.33	27.16	592	0.49	22.57	υ C2–C3(14)
55	536w	535w	528	4.67	8.29	531	5.13	9.50	τ H13C2C1C9(10) + τ C4C9C3O14(16) + τ C9C4C3O14(41)
56			521	23.61	23.56	526	25.38	22.55	υ C2–C3(21) + δC4C3O14(31) + δC6O16C17(10)
57	487m		473	4.01	8.01	477	4.14	7.52	δC6C7C8(11) + δC5C6C7(10)+ δ C3C4C9(18) + δ C7O21C22(13)
58		455w	457	0.24	19.61	462	0.22	19.79	τ C6C5C4C9(11) + γ O16C5C4C6 + γ O21C6C8C7(29)
59			435	10.99	50.14	438	10.59	46.89	υ C1–C9(13) + δC4C9C8(20) + δC7O21C22 (21)
60		366w	370	4.08	14.00	375	4.26	13.54	τ O6C5C4C9(22) + γ O16C5C7C6(11) + τ C1C9C4C5(31)
61			350	0.57	179.61	353	0.56	145.42	δ O6C5C4C9(20) + δC6O16C11(27)
62		293w	273	1.57	85.27	276	1.62	69.10	δC6C7C8(15) + δC4C3O14(16) + δC5C4C9(10) δC6O16C17(12) + δC7O21C22(16)
63			268	0.15	0.43	274	0.15	0.46	τ H18C17O16C6(16) + τ H23C22O21C7(52)
64		213m	237	0.04	12.36	245	0.02	10.99	τ H18C17O16C6(65) + τ H23C22O21C7(19)
65			197	0.35	44.24	201	0.38	40.28	τ H23C22O21C7(19) + τ C4C9C8C7(28)
66		185vw	185	1.59	62.85	188	1.72	51.94	δC6C7O21(50) + δC7C6O16(13+δ C7O21C22(19))
67		150vw	169	5.18	51.48	170	5.38	40.72	δC7C6O16(34) + δC3C4C9(17) + δC6O16C17(18)
68			144	0.33	2.04	146	0.31	0.37	τ C6C5C4C9(11) + τ C5C4C9C8(16) + τ C7C6O16C17(22) + τ C1C9C4C5(25)
69			130	2.62	10.88	131	2.72	13.81	τ C8C7O21C22(51) + τ C9C4C3O14(11) + γC3C5C9C4(14)
70		84m	84	1.98	3.11	83	4.15	4.72	τ C7C6O16C17(39) + τ C3C2C1C9(31)
71			74	6.55	2.98	72	4.70	2.88	τ C7C6O16C17(21) + τ C8C7O21C22(13) + τ C3C2C1C9(38)
72			64	2.09	53.21	65	2.29	54.46	τ C4C9C8C7(10) + τ C5C4C9C8(38)

IR _int_ - IR intensity; Ram_Int_ - Raman Intensity; Kmmol^−1^ w-weak; vw-very weak; s-strong; vs-very strong; m-medium; br, sh-broad, shoulder, υ - stretching; υ_sym_ – symmetric stretching; υ_asy_-asymmetric stretching; δ-in plane bending; γ-out-of –plane bending; τ-torsion.

#### 3.5.1 C–H vibrations

In this work, the assignment of C–H vibrations in 5,6-DMI molecule is easy task 5,6-DMI has two C–H moieties i.e.C5–H15 and C8–H26 units. Absorption values denote the occurrence of C–H stretching vibration falls between 3100-3000 cm^−1^ [31] in aromatic heterocyclic ring. In the FT-IR spectrum of 5,6-DMI, a very weak band observed at 3058 cm^−1^ and medium strong band in FT-Raman at 3059 cm^−1^ attributed to C–H stretching vibration. The wavenumber computed of this mode are at 3104 and 3096 cm^−1^(mode nos.1 and 2) by CAM-B3LYP/6-311G(d,p) method are assign to above said mode with TED contribution of 99% as shown in Table 3.

In 5,6-DMI, the C–H in-plane bending vibrations noted as strong to weak band in FT-IR spectrum at 1212, 1145 cm^−1^ and 1213 cm^−1^ as weak band in FT-Raman spectrum [31]. The wavenumber computed for this mode are 1240 and 1160 cm^−1^(mode nos.30 and 35) by CAM-B3LYP/6-311G(d,p) method with TED contribution of ∼20 % as shown as mixed mode in Table 3.

The C–H out-of-plane bending vibrations seem to be strongly coupled with other vibrations and appear in the range of 1000–750 cm^−1^ [31]. The aromatic C–H out-of-plane bending vibrations in 5,6-DMI are assigned at 851, 815 cm^−1^ in FT-IR spectrum and 816 cm^−1^ in FT-Raman spectrum is good correlate with B3LYP/6-311G(d,p) method at 873-812 cm^−1^ (mode nos. 45–47) with TED contribution of ∼77 %.

#### 3.5.2 CH_2_ group vibrations

Spectral studies shows, normally the asymmetric stretching vibrations for CH_2_ (methylene) group falls around 3000-2900 cm^−1^, while CH_2_ (methylene) symmetric stretching vibrations appear between 2900 - 2800 cm^−1^ [32,33]. Spectra of 5,6-DMI the predicted stretching mode (asymmetric and symmetric) of CH_2_ group by B3LYP/6-311G(d,p) method at 2992, 2956, 2954 and 2930 cm^−1^ (mode nos. 5, 8, 9 and 10) corresponds to the stretching modes of C1H_2_ and C2H_2_ units. The bands at 2983, 2953, 2925 cm^−1^ in FTIR spectrum and 2956, 2928 cm^−1^ in FT-Raman spectrum are assigned to CH_2_ antisymmetric and symmetric stretching vibrations with TED contribution of ∼94–98%.

It is well known that, CH_2_ bending vibrations are found between 1450-875 cm^−1^. The CH_2_ scissoring vibrations for cyclohexane found to be a medium intense IR band around 1450 cm^−1^ [34]. In the presence study of 5,6-DMI, the scaled wavenumber at 1443 and 1413 cm^−1^ (mode nos. 21 and 24) calculated by CAM-B3LYP/6-311G(d,p) method are assigned to CH_2_ scissoring modes of C1H_2_ and C2H_2_ units respectively. The recorded spectra in FT-IR at 1441, 1407 cm^−1^ and 1411 cm^−1^ in FT-Raman spectrum are attributed to CH_2_ scissoring vibrations. The computed wavenumber by CAM-B3LYP/6-311G(d,p) method at 1306-1258 cm^−1^(mode nos.27-28) are assign to CH_2_ wagging vibration of C1H_2_ and C2H_2_ units with TED contribution of ∼30%. The CH_2_ twisting vibrations observed in FT-IR spectrum at 1191 cm^−1^ and FT-Raman spectrum at 1195 cm^−1^.

#### 3.5.3 C=O vibrations

Absorption bands of C=O bond is seems to be strong band because of large change in the dipole and to be relatively free from other vibrations. Presence of large change in the dipole moment between carbonyl carbon and oxygen all carbonyl compounds show a very intense and narrow peak in the region 1800-1600 cm^−1^ [35]. In title compound, the C3 = O14 (keto group) stretching vibrations are recorded in FT-IR and in FT-Raman spectrum at 1703 and 1692 cm^−1^ as very strong band respectively. The computed wavenumber is 1722 cm^−1^ (mode no.13) with TED contribution of 88% correlate with related molecule [35]. The C3 = O14 in-plane and out-of-plane bending vibrations are computed by B3LYP/6-311G(d,p) method are at 521 and 370 cm^−1^ (mode nos.56 and 60) as shown in Table 3.

#### 3.5.4 O–CH_3_ vibrations

The methoxy groups (alkoxy) make the aromatic ring more electron rich and its vibrations are affected by number of interactions namely inductive effects, inter-molecular hydrogen bonding and Fermi resonance, etc., [34].

The organic functional groups, the oxygen containing functional group like alkoxy (RO-) group present in numerous aromatic compound. A methoxy group is a good donar of its loan pair of electrons which is used in conjugation. In methoxy group, presence of highly electronegative oxygen atom, it can inductively withdrawn electrons through σ bonds.

A key observation was made that, methoxy group normally affects the pi electron cloud of the ring strongly. A functional group having oxygen atom near to alkyl group cause back donation and induction effects, so it alter the position of C–H stretching and bending modes [34]. Normally, IR and Raman spectrum of methoxy group vibrations are observed as intense band, with expected large variation. The reason for this deviation is due to electronic effect. This electronic effect which leads the molecule to have a spectral values deviated from the expected values has been as reported earlier [36].

The title compound 5,6-DMI is a non-planar molecule with one methoxy carbon atom lies in the plane and other out of the ring which may implies that two methoxy groups does not form any bonding between them. In our study a very weak bands recorded in FT-IR at 3024, 2972 cm^−1^ and medium band in FT-Raman spectrum at 3024 cm^−1^ are attributed to C17H_3_ and C22H_3_ stretching mode of asymmetric and symmetric vibrations. The wavenumbers theoretically computed for this mode are at 3035, 3033, 2963, 2961, 2906 and 2904 cm^−1^(mode nos.3, 4, 6, 7, 11 and 12) by B3LYP/6-311G(d,p) method with TED contribution of ∼94%.

In earlier studies shows that the O–CH_3_ stretching vibration is falls near ∼1040 cm^−1^ for anisole [37] and 1000-100 cm^−1^ for its derivatives. In 5,6-DMI, the methoxy group (O–CH_3_) stretching mode are appear as very strong to weak band observed in FT-IR spectrum at 1039, 984 cm^−1^ and very weak band at 1035, 990 cm^−1^ in FT-Raman spectrum. The results are shows good agreement with computed wavenumber 1028 and 1021 cm^−1^ (mode nos. 40 and 41) by B3LYP/6-311G(d,p) method and coincides with experimental results with TED contribution of ∼30%.

The assignment of the C–O–CH_3_ angle bending mode vibration for anisole is falls around 300 cm^−1^ was reported by Owen and Hester [38]. Campaqnaro and Wood [39] assigned the wavenumber at 421 cm^−1^ for p-methoxy benzaldehyde for the above said vibrations. In accordance with above literature data, assignments of C–O–CH_3_ angle bending mode vibrations assigned theoretically by B3LYP/6-311G(d) method at 370 cm^−1^ (mode no.60) shows good agreement with recorded FT-Raman band at 366 cm^−1^ with TED contribution of 22 %. The torsional mode of for anisole can be obtained around 100 cm^−1^. In 5,6-DMI molecule O–CH_3_ torsional mode was calculated at 84 and 74 cm^−1^ (mode nos. 70–71) corresponds to O17–C17H_3_ and O21–C22H_3_ by B3LYP/6-311G(d,p) method.

#### 3.5.5 Ring vibrations

The aromatic ring in a structure is easily determined by C–C and C=C stretching vibrations and we expected ring carbon–carbon stretching falls between 1625-1430 cm^−1^. By Varsanyi et al. [31], the C–C stretching bands falls in variable intensity at 1625–1590, 1590–1575, 1540-1470, 1465-1430 and 1380-1280 cm^−1^. In 5,6-DMI, wavenumber computed at 1584, 1571, 1486, 1346, 1290, 1252, 1097-1021 and 965-944 cm^−1^ (mode nos.14, 15, 16, 26, 27, 28, 39–41 and 43–44) by B3LYP/6-311G(d,p) method for both the ring system. In FT-IR spectrum the wavenumbers recorded at 1590, 1501, 1359, 1316, 1261, 1039, 984, 960 and 897 cm^−1^ are assigned to stretching mode of C–C vibrations. Band due to C–C stretching were recorded in FT-Raman spectrum are at 1592, 1500, 1311, 1035 and 990 cm^−1^. Based on the PED table these vibrational modes are mixed mode combination with C–H in-plane bending, CH _2_ wagging and torsion vibrations in this region. The in-plane deformation vibrations are obtained at higher wavenumbers when compared with to out-of-plane bending vibrations. In this molecule, the bands observed at 748, 709, 640, 603, 577, 536, 455 and 487 cm^−1^ in FT-IR are assigned to C–C–C deformation vibrations of the phenyl and five membered cyclopentanone ring. The C–C–C vibrations in FT-Raman spectrum is at 816, 752, 644, 605, 535 and 366 cm^−1^. The theoretically computed values at 738, 712, 695-528, 473-370 cm^−1^ (mode nos. 49, 51–55, 57–60) by B3LYP/6-311G(d,p) method.

### 3.6 ^13^C and ^1^H NMR spectral analysis

The chemical shifts values in NMR spectroscopy prove a powerful tool for structural identification and confirming the structure of organic and inorganic molecules. It gives the local structure surrounding the nucleus of interest. The isotropic chemical shifts used to determine the structure of the molecular and magnetic properties [40]. The recorded ^13^C and ^1^H NMR spectra of 5,6-DMI is shown in Figure S1 (a) and (b).

The DFT computations are seems to be fast also it applicable especially for large molecule. The most popular techniques is GIAO method for predicting isotropic chemical shift for large molecule [40]. The computed NMR chemical shift of 5,6-DMI predicted by B3LYP/6-311G(d,p) and CAM-B3LYP/6-311G(d,p) method with experimental recorded NMR chemical shifts are tabulated in Table 4. Normally, ^13^C NMR signals are much weaker than the ^1^H NMR signals and its chemical shift found around >100 ppm [41, 42]. In our present investigations, the 5,6-DMI ^13^C NMR chemical shift values falls with the above literature data expect carbon atom in methoxy group and five membered carbon atoms. In 5,6-DMI, the signal observed at 36.05, 25.06 and 55.85, 55.52 ppm in ^13^C NMR spectrum is attributed to C2, C1(five membered) and C17, C22 (methoxy carbon atom). The calculated chemical shifts of above said ^13^C NMR shift are at 37.75, 27.0 and 57.21, 55.0 ppm as shown in Table 4. The signals for phenyl ring carbons were observed at 129.8, 108.2, 148.9, 155, 103.6, 150.1 ppm in ^13^C NMR spectrum for the title molecule. The additional signals (triplet at 39.77, 39.50, 39.22 ppm) arise due to solvent peak. The signals of the aromatic protons (protons at phenyl ring) were observed as a singlet at 7.08 and 7.03 ppm corresponding to H26 and H15 unit. The ^13^C NMR chemical shift for C3 = O14 is recorded at 205.64 ppm [43] is correlate with 206.6 ppm by CAM-B3LYP/6-311G(d,p) method.

**Table 4:** Experimental and theoretical chemical shifts (^1^H and^13^C) of 5,6-DMI by DFT method.

Atom position	Expt	B3LYP 6-311G(d,p)	CAM-B3LYP 6-311G(d,p)
C1	25.06	27.0	22.47
C2	36.05	37.75	33.68
C3	205.64^a^	206	206.6
C4	129.8	132.60	130.29
C5	108.2	109.59	103.68
C6	148.9	155.08	152.23
C7	155.0	159.12	159.560
C8	103.6	108.83	106.49
C9	150.1	155.37	153.29
H10	2.98	3.015	2.86
H11	2.97	3.012	2.86
H12	2.56	2.48	2.38
H13	2.54	2.49	2.38
H15	7.08	7.53	7.09
C17	55.85	57.21	50.51
H18	3.78	3.86	3.60
H19	3.78	3.74	3.60
H20	3.78	4.28	4.11
C22	55.52	55.0	50.68
H23	3.85	3.88	3.61
H24	3.85	3.79	3.61
H25	3.85	4.27	4.17
H26	7.03	7.07	6.77

a Taken from Ref [43].

In 5,6-DMI, the ^1^H chemical shifts were recorded and calculated for protons of CH_3_ groups. The values are quite low. All ^1^H chemical shift values are found to be ≤3 ppm [44] due to the shielding effect. In 5,6-DMI molecule, the methoxy protons at the C17 and C22 appears as singlet with three proton integral as evident from recorded spectrum at 3.78 and 3.85 ppm respectively, shows good correlation with computed shift at 3.86, 3.74, 4.28 and 3.88, 3.79, 4.27 ppm as shown in Table 4. The five membered cyclopentanone moiety, the hydrogen atoms are falls at 2.54–2.98 ppm as shown in Table 4.

### 3.7 UV-vis spectra and electronic properties

**Figure 6:**
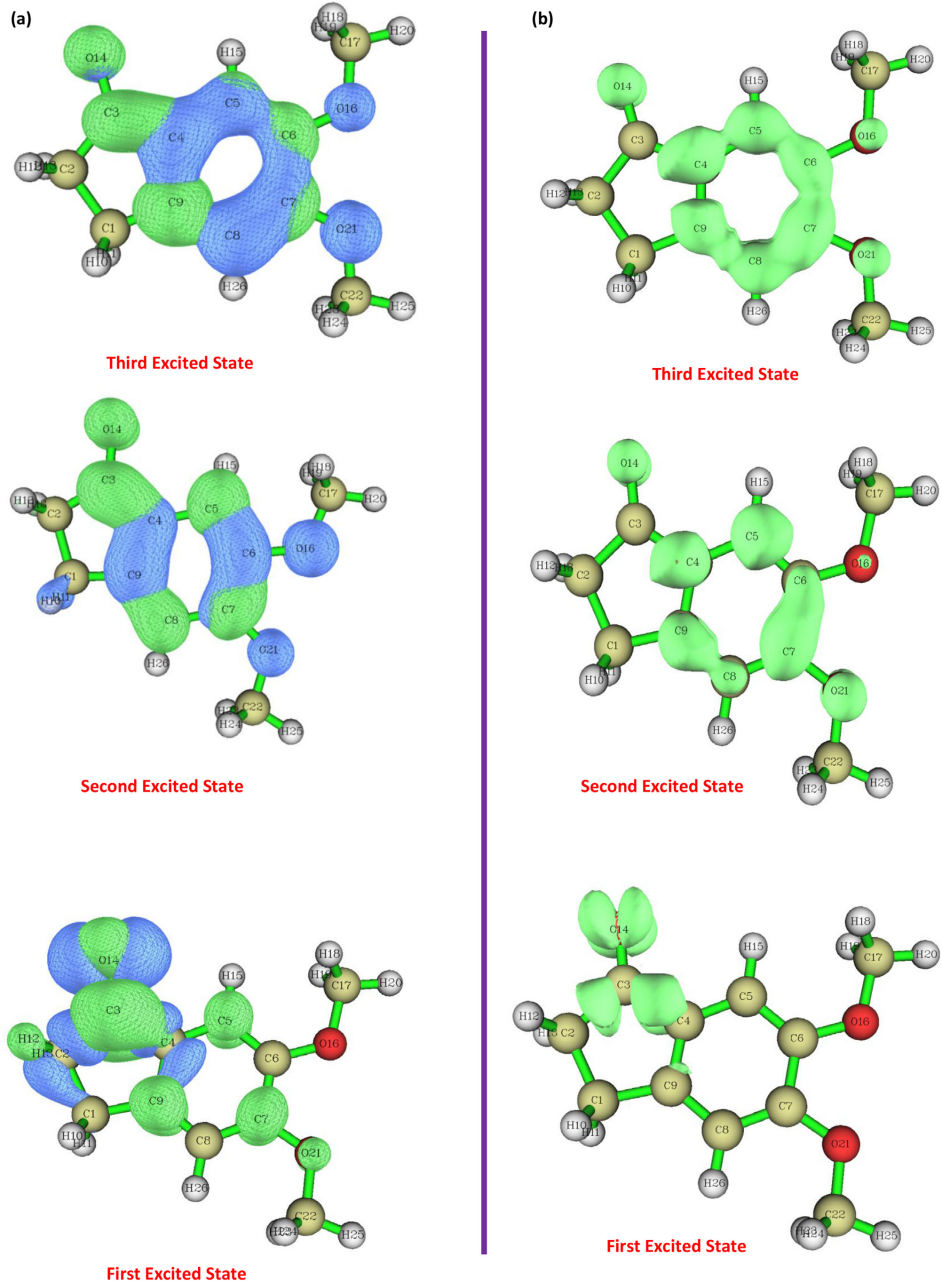
Electron – hole distribution (a) and electron – hole overlap (b) for three excited states of 5,6-DMI molecule.

In order to completely understand the molecular electronic transition and elemental electronic transition of 5,6-DMI molecule, TD-DFT [21] and IEF-PCM model [22] calculations were computed for gas and ethanol phase and results are tabulated in Table 5. The UV-Vis spectra of 5,6-DMI was recorded using ethanol solution are shown in Figure 3. Ethanol was used as a solvent due to its no significant interference in UV-Vis spectroscopy. By analyzing the recorded UV-Vis spectrum of the title compound, it is well noted that two intense bands are observed at 311 nm and 266 nm represent the transition from n→π∗. The electronic assignment correspond to the first transition is n→π∗ (nonbonding electron pair to antibonding orbital), The n→π∗ transition leads charge formation, so n system become deficient in electron while π∗ system acquire an extra electrons causes separations of charges in the systems to stabilize π∗ orbital [45]. The stimulated ethanol phase spectra are shown in Figure S6. The frontier molecular electron density denotes the reactive site of π electron density among the delocalized conjugated system in the molecule [46]. The HOMO-LUMO is found to be small if the molecule has conjugated system which leads to have more chance of intermolecular charge transfer from end-copping electron donor group to electron acceptor through π conjugated system [47]. The HOMO and LUMO energy are -5.95 and -1.27 eV, energy gap between the orbitals is 4.64 ​eV. The energy gap evident that 5,6-DMI provide the bioactivity property through intramolecular charge transfer [48, 49]. The maximum absorption wavelength assigned to possible transition from HOMO-2→LUMO (89%), HOMO→LUMO (87%) and HOMO-1→LUMO (56%), HOMO→LUMO+1 (34%). The HOMO plot shows charge density mainly located on C=O and methoxy group, LUMO plot is localized on entire ring system i.e the electron charge density distribution is transferred from C=O and methoxy group to the ring system. Table 6 shows the charge transfer length (D), Δr and excitation energy (E) electron – hole distribution (S) for 5,6-DMI molecule for three excited state which was computed by Multiwfn program package [24] and the illustrated diagram are shown in Figure 6 (a) and (b). We want to mention that the distance between the centroid of the hole to the electrons in a molecule gives the charge transfer length (D), the larger the values of D will leads to movement of charge density from one place of the molecule to the other place. The computed charge transfer length seems to be higher for first excited state 1.0557 Å when compare to others (second and third excited state 0.8698 Å and 0.8779 Å). The distribution of hole in the molecule is more, when compare to electron which is denoted by blue and green colour respectively, this indicate that electrons distribution is more possible in the ring and group during transition. The Δr indicates the quantitative indication of electron excitation mode, from Table 6 the first excited state having the highest Δr value of 1.9754 followed by second excited state (1.3621) and third excited state (1.1391). The excitation energy increases as the excited state increases as shown in Table 6.

**Table 5:** Experimental and calculated absorption wavelength and oscillator strengths of 5,6-DMI using the TD-DFT method at B3LYP/6-311G(d,p) and CAM- B3LYP/6-311G(d,p) method.

B3LYP/6-311G(d,p)		CAM-B3LYP/6-311G(d,p)		Expt. (nm)	Assignment	In Solvent aMajor Contribution (≥10%)
Wavelength λ (nm) Ethanol Phase	Oscillator Strength (f)	Wavelength λ (nm) **Cal.** Ethanol	Oscillator Strength (f)			
307.89	0.0001	290.68	0.0001	311	n→π∗	H-2 →L(89%)
295.23	0.2065	273.04	0.2449	266	n→ π ∗	H→L (87%)
253.86	0.1617	238.30	0.1491	228	n→ π ∗	H-1→L(56%), H→L+1 (34%)

a H-HOMO; L-LUMO.

**Table 6:** Overlap integral, Charge transfer length, Δr and excited energy for different excited states.

Excited state	Overlap integral of electron – hole(S)	Charge Transfer length (D) (Ǻ)	Δr (Ǻ)	Excitation energy (E) eV
1	0.24098	1.0557	1.9754	4.1382
2	0.50833	0.8698	1.3621	4.6266
3	0.60956	0.8779	1.1391	5.2670

### 3.8 Total, partial and population density of state

**Figure 7:**
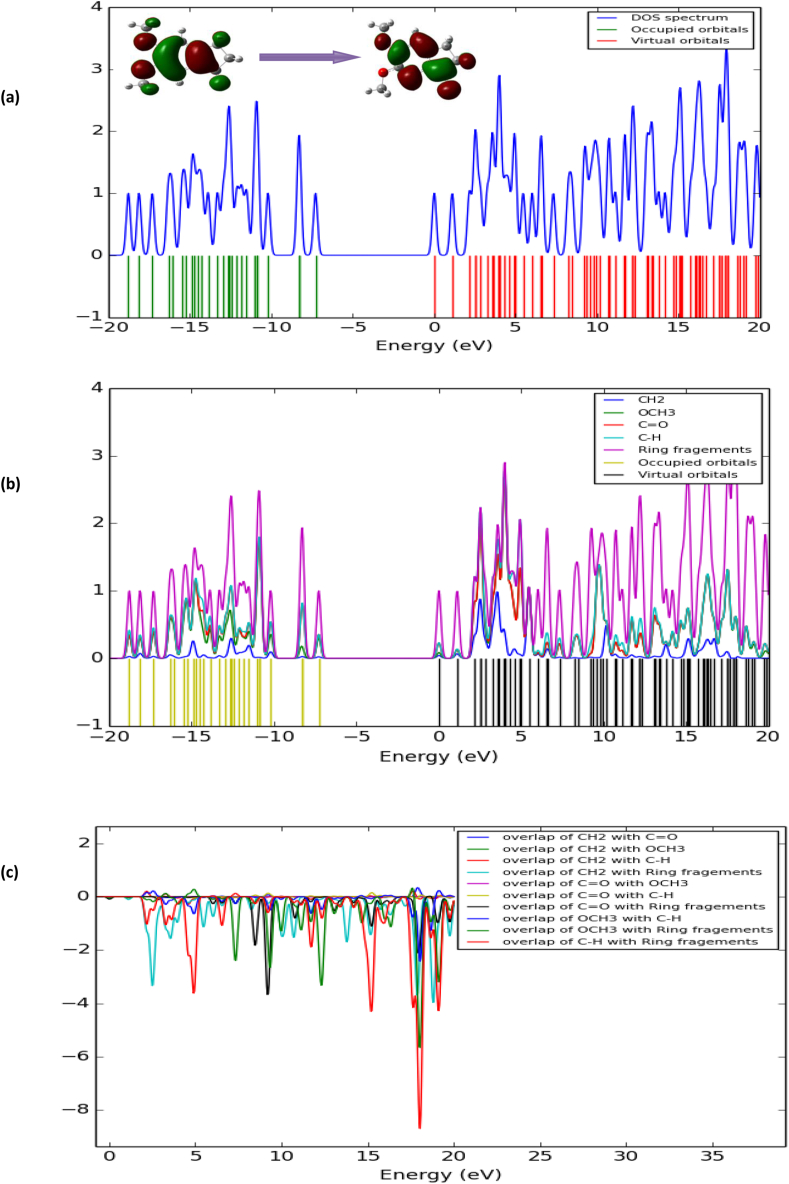
Density of state (a), partial density of state (b) and overlap population density of state (c) of 5,6-DMImolecule.

The aromatic conjugated orbital's show quasi degenerate energy level in the boundary region. The Total Density of State (TDOS) and Partial Density of State (PDOS) are shown in Figure 7 (a), (b) and (c) using Gauss Sum 2.2 program [23]. The PDOS represent the fragment composition in molecular orbital, the HOMO orbitals are localized in the C=O, OCH_3_, CH_2_ (28% + 2%+65% = 100%) and LUMO orbital spread on the C=O, OCH_3_, CH_2_ and ring fragment as (14% + 4 % + 4% + 77 % = 99%).

The Electronegativity (χ), Chemical hardness (η), Softness(S), Chemical Potential (μ) and Electrophilicity index (ϖ) are computed for gas phase and ethanol phase by B3LYP/6-311G(d,p) and CAM-B3LYP/6-311G(d,p) method are shown in Table S4. The electronegativity measure the negative of electronic chemical potential. Chemical softness represent inverse of chemical hardness, Chemical hardness represent hard to accept electron easily. As we seen from Table S4 the magnitude of the softness are shown in equal scale which implies maximal electron transfer from between electron donar to acceptor. The electrophilic index and other parameter and increase as the solvent phase is changes from gas phase.

### 3.9 Molecular electrostatic potential (MEP)

The MEP illustrate the reactivity for a hydrogen bonding interaction, electrophilic and nucleophilic attack [50, 51] of the molecule three dimensionally. The study provides a visual method and correlate with dipole moment, electronegativity and partial charges.

The colour scheme used to denote MEP surface where increase from red to blue colour. The colour range falls between red to blue colour (−0.0592 a.u to +0.05929 a.u). The red colour show strongest repulsion and blue colour refer strong attraction. The sliced map of 5,6-DMI in 2D MEP is shown in Figure S7 (a) and (b), this picture provide important data about MEP distribution. The Oxygen and C=O group have been depicted as electron rich region and all hydrogen atoms corresponds to electron deficient region. The MEP surface for 5,6-DMI was illustrated by B3LYP/6-311G(d,p) method as shown in Figure S7 (a). The negative electrostatic potential situated over the carbonyl and methoxy group atoms. The possible nucleophilic site is found near all ring carbons and hydrogens. The contour electrostatic map of 5,6-DMI were predicted by B3LYP/6-311G(d,p) method are illustrate in Figure S7 (b).

### 3.10 Reduced density gradient

Johnson and Co-Worker [52] derived the theory to explain weak interaction in real space can be found by finding the electron density in the molecular system. The RDG is a dimensionless quantity and its first derivation has been written as Eq. (3).

(3) $$RDG=\left(r\right)=\frac{1}{{2\left(3\pi r^{2}\right)}^{\frac{1}{3}}}\frac{|{\text{Δ}}^{2}\rho \left(r\right)|}{\rho {\left(r\right)}^{\frac{4}{3}}}$$

**Figure 8:**
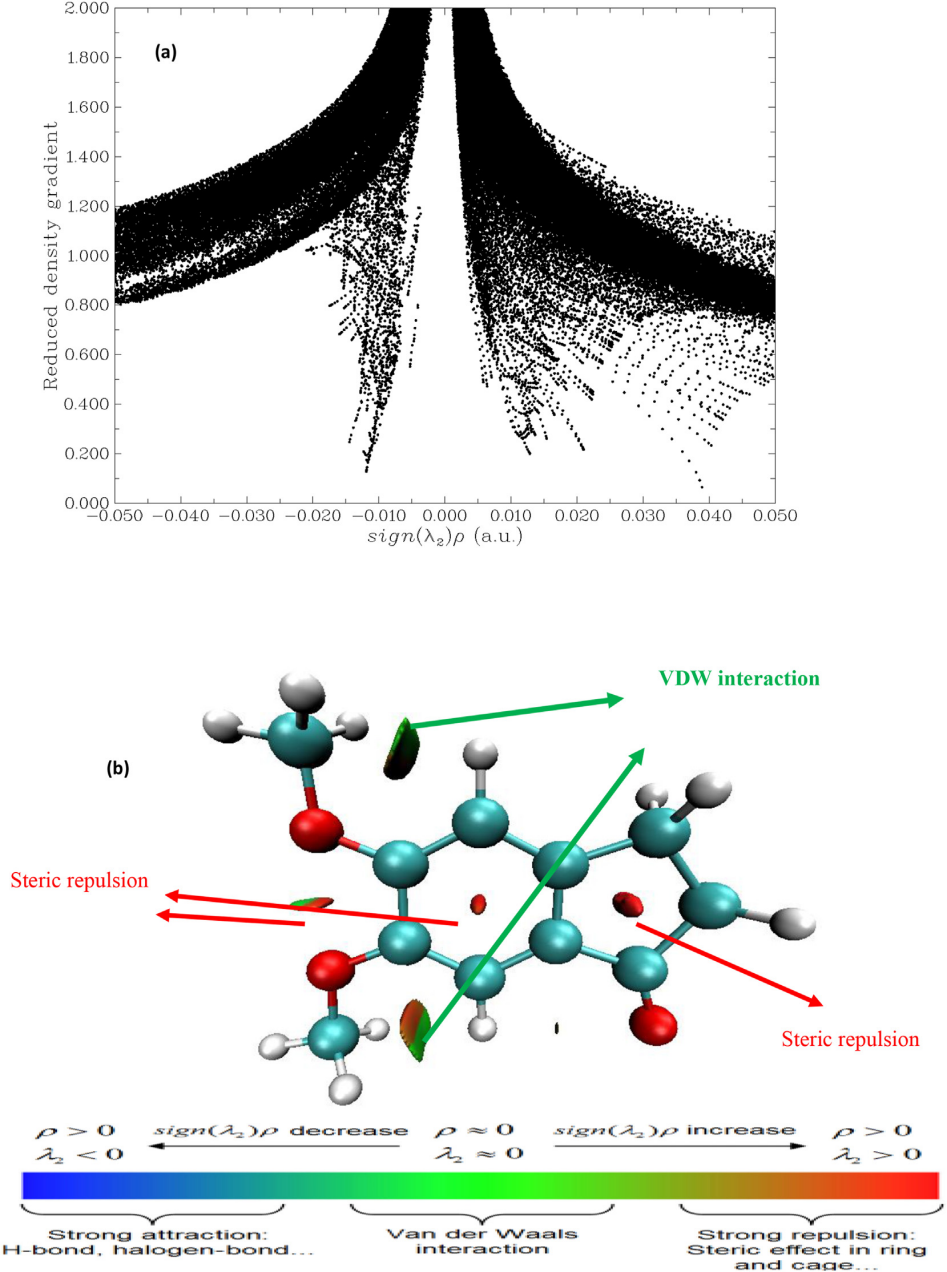
The Reduced density gradient (a) and colour (blue-green scale) surface (b) of 5,6-DMIaccording to λ_2_.

The weak interaction is examining by finding low electron density region. The Figure 8 (a) and (b) show the electron density ρ(RDG) vs multiplied by sign of λ_2_. The λ_2_ is help to discriminate bond (λ_2_ < 0) from non bonding (λ_2_ > 0). The Multiwfn program [24] and VMD program [25] is used to calculate RDG calculation. The RDG = 0.10 lines in the RDG diagram show the molecule have attraction and repulsion spike. The negative value of (λ_2_) ρ denote the strong attraction and positive value of sign (λ_2_) ρ denote the strong repulsion. The value near to the zero denotes very weak Van Der Waals (VdW) interactions. The colour indicate in blue to red denote stronger to repulsive interaction in the molecular system. The interaction region marked in green evident the presence of VdW interaction in our molecule, the VdW present in between OCH_3_…. H in the oxygen atom show the strong steric effect in title molecule and it is shown by red colour.

### 3.11 Electron localization function (ELF)

**Figure 9:**
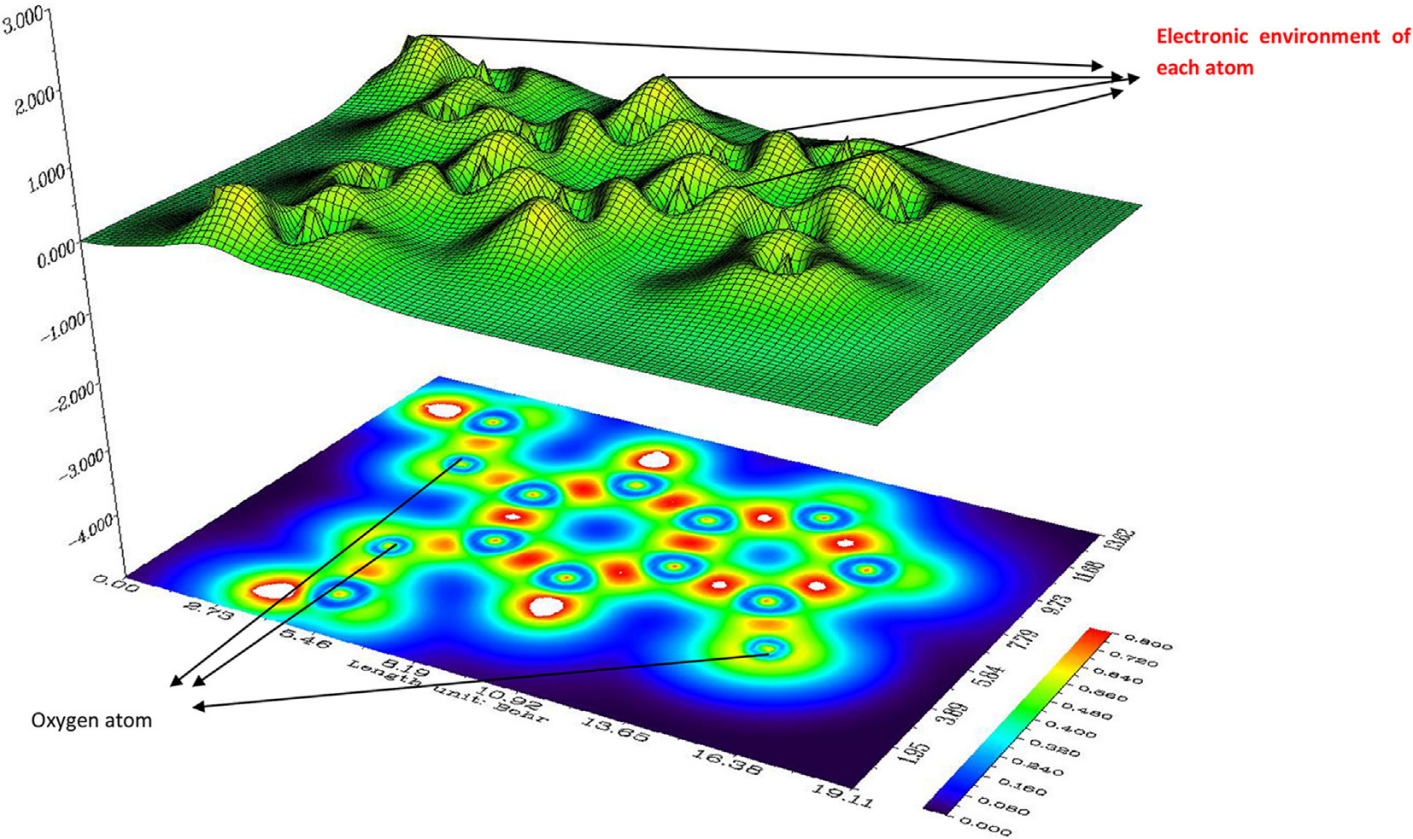
3D Electron localization function diagram 5,6-DMI.

ELF is used to locate the electron pair in the molecule and denoted a scalar function η(r) and it also related to Fermi hole curvature [53]. It computes excess kinetic energy density due to Pauli relation. Multiwfn software [24] are used to draw ELF diagram are shown in Figure 9. This analysis use gradient field to locate attractors and basins. The colour representation of 3D plot of ELF are shown in Figure 9, the various colours are: blue region are charge depletion; orange region denote charge accumulation; green region denote weak interaction in the molecule. In 5,6-DMI the carbon atoms shows charge depletion and in between the two carbon atom charges accumulated are represented in orange colour.

**Figure 10:**
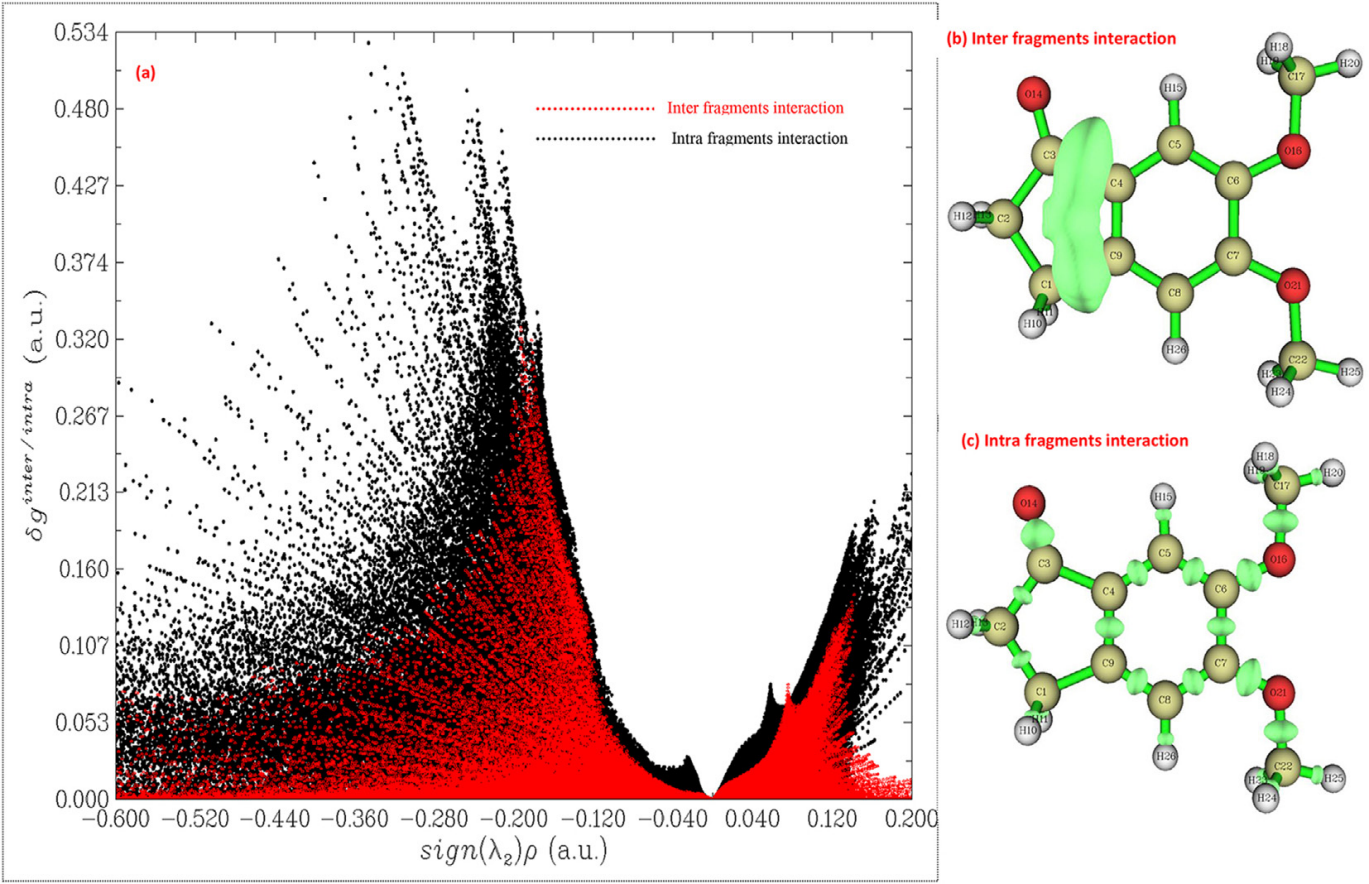
Intra and inter fragments interaction scattering graph (a), inter fragments interaction (b), intra fragment interaction (c) in the 5,6-DMI.

The Figure 10 (a), (b) and (c) shows the scatter graph of inter and intra fragments interactions of 5,6-DMI molecule, the red colour in the Figure 10 (a), (b) and (c) indicate the inter fragment interaction and black colour denote the intra fragment interaction. The negative sign of λ2∗ρ denote the attraction interaction due to hydrogen bond formation. The area at λ2∗ρ at +0.04, a small peak at a height of 0.053 indicate the strong repulsion and steric interaction in the 5,6-DMI molecule. The value of -0.040 indicated in the peak in terms of intra fragment interaction of 5,6-DMI due to chemical bond strong attractive inter molecule interaction. Figure 10 (a) is the intra fragment interaction with VdW interaction (with isosurface value 0.2 to 0.02), The isosurface due to inter and intra fragment are shown in Figure 10 (b) and (c) (with 0.2 to 0.15).

### 3.12 Drug likeness

The molecule 5,6-DMI has been checked for drug likeness parameter to examine the possible potential to play vital role in pharmaceutical product. So that the drug likeness parameter are summarized in Table S5, contain the predicated value of hydrogen bond donars (HBD), hydrogen bond acceptors (HBA), number of rotatable bonds, Alog P, polar surface area (PSA) and molar refractivity for 5,6-DMI molecule. According to Lipinski's rule of five [54], the parameters are falls within the range of HBD and HBA is less than 5 and 10 respectively, the rotatable bond in the molecule is allowable is within 10. Alog P value(indicating the hydrophobic/lipophilic character of molecule) is less than 5 as denoted in the Lipinski's rule of five. The number of hydrogen bond acceptor is 3, the Alog P value is 2.21 and molecular refractive is 52.47 which is fall between the allowed range of 40–130 for pharmaceutical drug as reported [55, 56]. Based on the above said data, 5,6-DMI have been consider as a pharmaceutical active compound.

### 3.13 Molecular docking study

**Figure 11:**
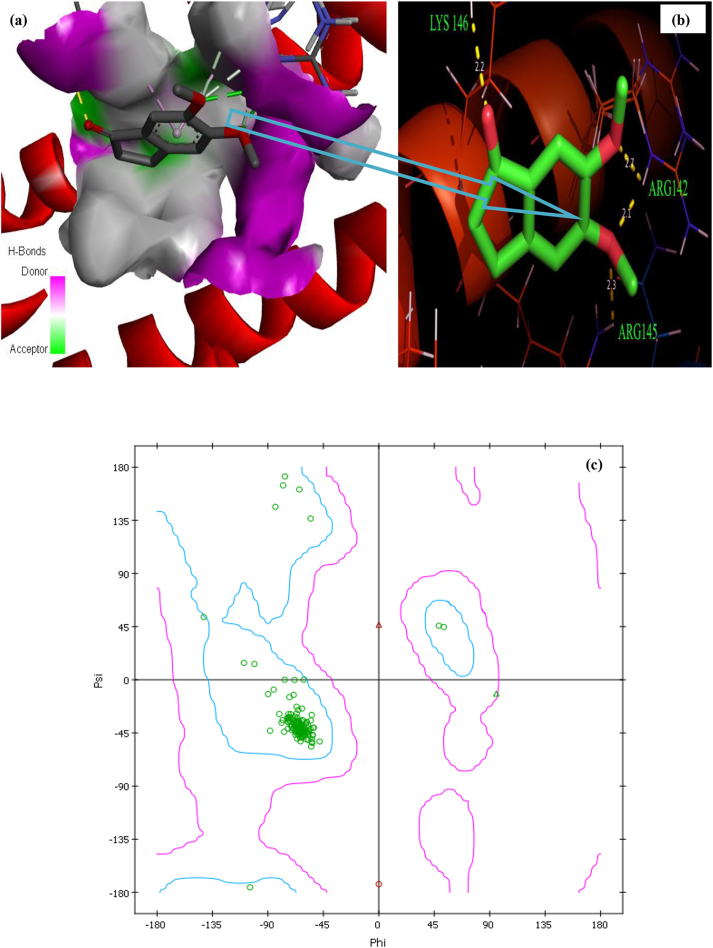
Protein-ligand interaction in 3D form (a), protein-ligand hydrogen bond distances (b), Ramachandra Plot (c) of 5,6-DMI with apoE protein.

The indanone derivatives beneficial pharmacological properties and it's useful for Alzheimer's disease treatment [57]. Apolipoprotein E (apoE) is a plasma protein and found to be an important lipid-transport protein in human plasma and brain. apoE is the major genetic risk factor in neurodegenerative, heart and Alzheimer's diseases [58]. In this present study, we have selected the apoE target protein to evaluate the biological efficiency of the title ligand. The apoE protein was readily available from RCSB protein data bank (PDB ID: 1B68) with a resolution of 2.0 Å [59]. The protein was prepared by remove the co-crystallized ligand and water molecules. All hydrogens were added and Kollman charges were assigned to all atoms of the protein using AutoDock Tools, graphical user interface provided by MGL Tools 1.5.6 program [60, 61]. The ligand structure was taken from minimized energy (C1 conformer) using Gaussian 09W program [15]. All residues of the target protein were mapped with grid box size of 82 Å × 82 Å x 82 Å points using AutoGrid 4 [62,63]. The binding pose diagram is shown in Figure 11 (a) and (b) this denote the possible formation of contacts with the amino acid residue such as LYS146, ARG142 and ARG145 with bond length of 2.2, 2.7, 2.1, 2.3 Ǻ are shown in Table 7a and Table 7b. The Discovery studio visualize software [64] is used to draw Ramachandra plot as shown in Figure 11 (c). The plots may helpful to show the allowed region by drawing plot between ψ and φ residue of the amino acid. From Figure 11 (c) blue region point out the allowed region and it also indicate the binding energy strength seems to be high while docking. The docking analysis propose that 5,6-DMI molecule have the ability to show inhibitory activity in opposition to apoE protein.

**Table 7(a):** Molecular docking results of 5,6-DMI molecule with apoE protein targets.

Drug	Protein ID	Binding energy (Kcal/mol)	Estimated inhibition constant Ki (μM)	RMSD
5,6-DMI	1B68	-4.24	777.57	42.02

**Table 7(b):** Summary of hydrogen bonding of 5,6-DMI molecule with different types of cancer protein targets.

Protein (PDB ID)	No. of hydrogen bond	Bonded Residues	Bond Distance
1B68	4	LYS146	2.2
		ARG142	2.7
		ARG142	2.1
		ARG145	2.3

### 3.14 Thermodynamic parameters

The several thermodynamical parameters such as Heat capacity, Entropy and Enthalpy were computed by B3LYP/6-311G(d,p) and CAM-B3LYP/6-311G(d,p) method and the obtained data are tabulated in Table S6–S7. In our study the thermodynamic function are found by varying the temperature from 100 ​K up to 700 ​K in the steps of 100 ​K. As we found that the molecular vibrational intensities increase with increase in temperature. We also calculate the correlation equation between the above said parameters by quadratic formula and corresponding fitting factors (R^2^) for the thermodynamic properties for B3LYP/6-311G(d,p) and CAM-B3LYP/6-311G(d,p) method are shown in Eqs. (4), (5), (6), (7), (8), and (9) below.

(4) $$C_{p,m}^{o}=3.14+0.172T-6.0\times 10^{-5}T^{2}\left(R^{2}=0.999\right)$$

(5) $$C_{p,m}^{o}=3.19+0.168T-6.0\times 10^{-5}T^{2}\left(R^{2}=0.999\right)$$

(6) $$S_{m}^{o}=55.12+0.2045T-5.0\times 10^{-5}T^{2}\left(R^{2}=0.999\right)$$

(7) $$S_{m}^{o}=55.124+0.201T-5.0\times 10^{-5}T^{2}\left(R^{2}=0.999\right)$$

(8) $$H_{m}^{o}=131.0+0.010T-6.0\times 10^{-5}T^{2}\left(R^{2}=0.999\right)$$

(9) $$H_{m}^{o}=132.8+0.010T-6.0\times 10^{-5}T^{2}\left(R^{2}=0.999\right)$$

As we seen from the correlation graph Figure S8 (a) and (b), the heat capacity, entropy and enthalpy increases as the temperature increases. This thermodynamic parameters are used for further analysis of 5,6-DMI. The value computed in Table S6–S7 are helpful to determine other thermodynamic energies by the relation of thermodynamic function and chemical reaction such as reaction coordinate according to second law of thermodynamic in thermochemistry and we also notify that all thermodynamic parameters are computed in gas phase only they could not be used in solution.

## 4 Conclusion

The conformation analysis suggests that the most stable of 5,6-DMI is C_1_ conformer by finding the lowest minimum energy. The geometrical parameters computed theoretically are in good correlation with XRD results. The carbon-carbon bond length of the six membered ring seems to be more coincide with XRD data when compare with five membered ring. From PES scan study the energy change related to rotation of OCH_3_ group denote, both OCH_3_ group orient in opposite plane correlate with C_1_ conformer. The influence of carbonyl and methoxy group to the vibrational frequencies of the 5,6-DMI have been discussed in detailed manner. NBO analysis revels the charge transfer lead to ICT between C=O, methoxy group and ring systems. The charge variation among the atoms is discussed in charge analysis. The NMR spectra were recorded and compared with computed values indicate all aromatic carbon and hydrogen chemical shift are found within range. The HOMO and LUMO energies evident the charge transfer occurs in the molecule. The MEP analysis evident that electron cloud distributed over the C=O and methoxy group and positive charge are surrounded over all hydrogen and ring carbon atoms. From RDG analysis, the presence of VdW and steric interaction are identified and it is found that the VdW interaction if found inbetween methoxy hydrogen and ring hydrogen atoms, and steric interaction is found in the ring system and in-between two methoxy oxygen atoms. The thermodynamic properties suggest that thermodynamical parameters increase with increase in temperature. The molecular docking results recommend that 5,6-DMI molecule might show inhibitor activity in opposition to apoE protein from the computed lowest binding free energies.

## Declarations

### Author contribution statement

S. Sebastian: conceived and designed the experiments; analyzed and interpreted the data; wrote the paper.

N. Sundaraganesan, S.Silvan: analyzed and interpreted the data; wrote the paper.

S. Sylvestre: conceived and designed the experiments; wrote the paper.

B. Karthikeyan: performed the experiments; analyzed and interpreted the data; wrote the paper.

### Funding statement

This research did not receive any specific grant from funding agencies in the public, commercial, or not-for-profit sectors.

### Data availability statement

Data included in article/supplementary material/referenced in article.

### Declaration of interests statement

The authors declare no conflict of interest.

### Additional information

No additional information is available for this paper.

## Appendix A Supplementary data

Table S5 Table S5

Table S6 _spl_ S7 Table S6 _spl_ S7
