# Supplementary figures and images for: Conformational analysis, molecular structure, spectroscopic, NBO, reactivity descriptors, wavefunction and molecular docking investigations of 5,6-dimethoxy-1-indanone: A potential anti Alzheimer's agent
Source: Heliyon. 2022 Jan 23;8(1):e08821. doi: 10.1016/j.heliyon.2022.e08821 (PMC8808071; doi:10.1016/j.heliyon.2022.e08821)

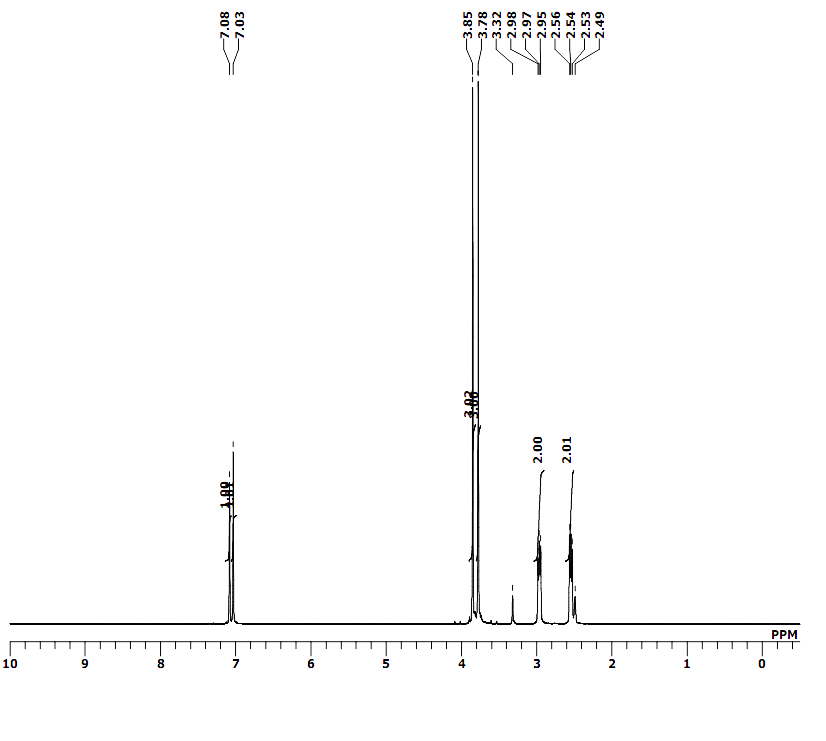


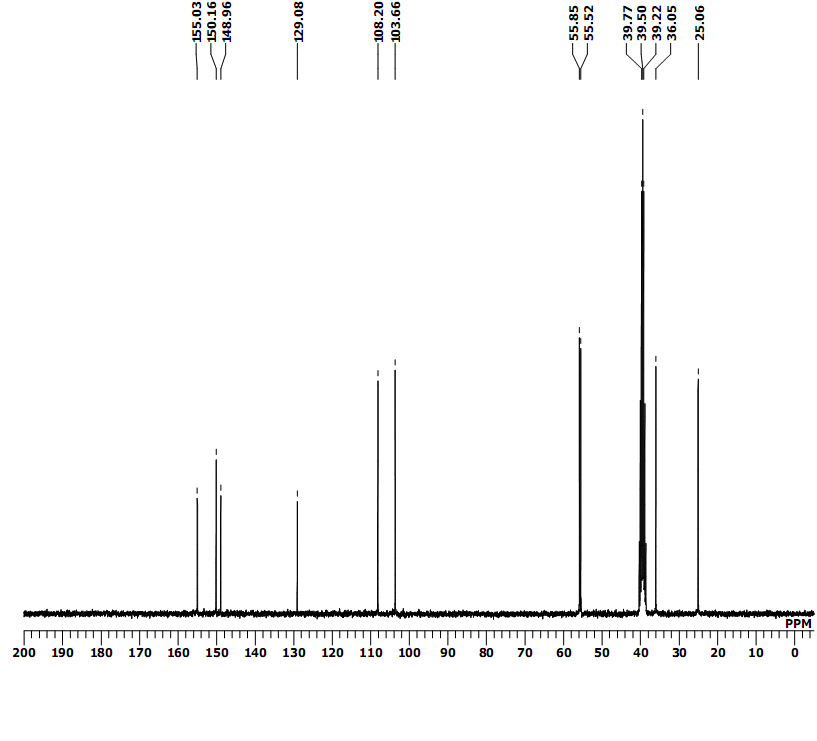

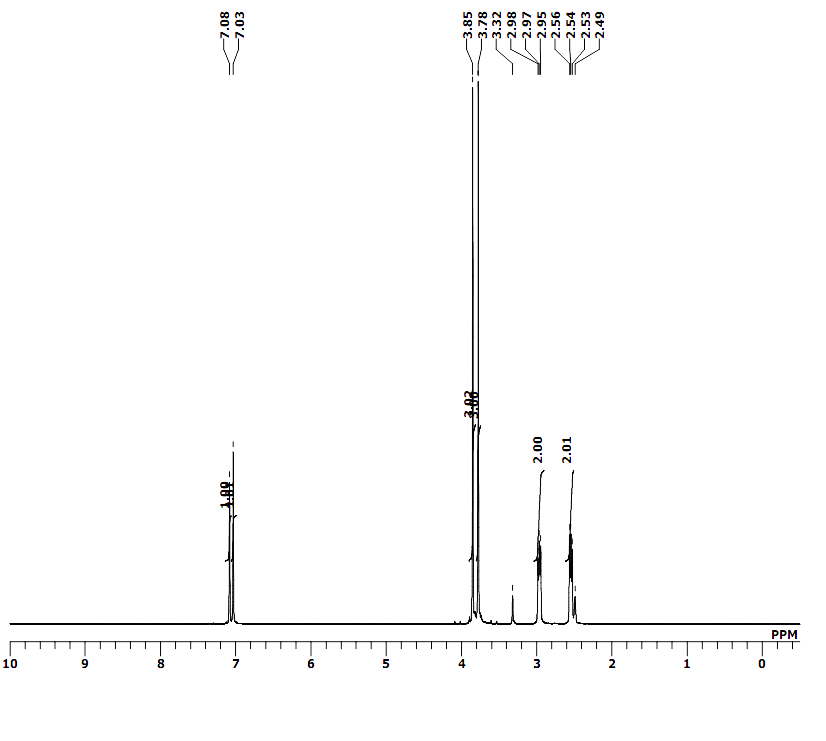


**Figure S1. 1H (a) and 13C (b) NMR spectrum of 5,6-DMI**

**(a)**

**(b)**

Supplement: Figure S1 [file mmc1.doc]

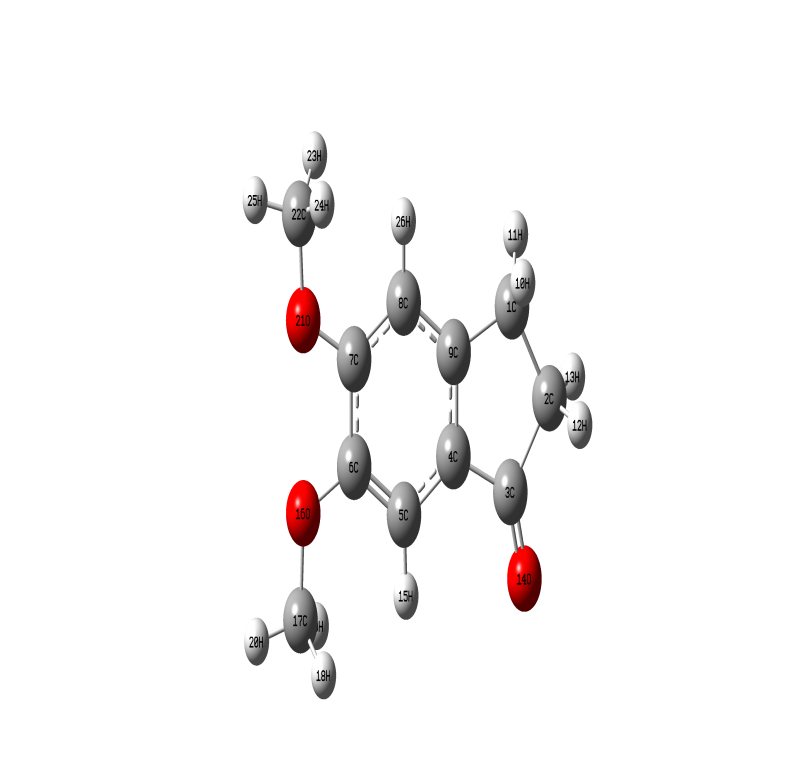

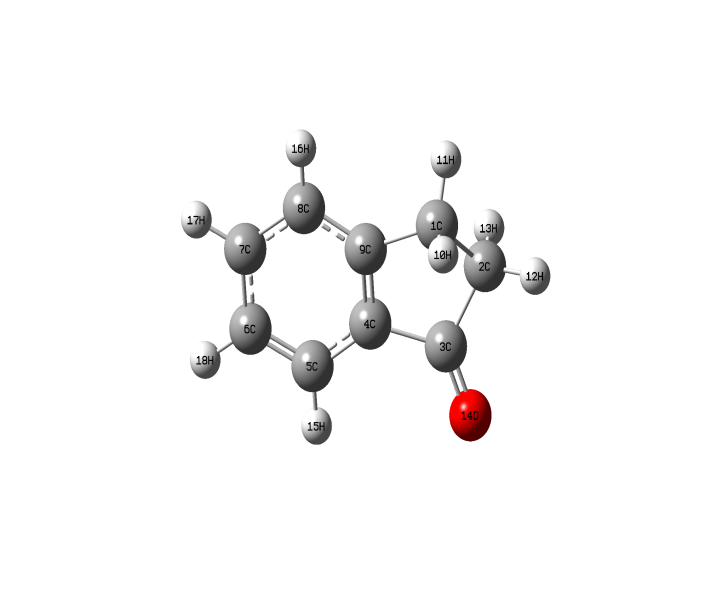


**(b)**

**(a)**

Charge (a.u)

Charge (a.u)

**Figure S3. NBO Charge analysis of Indanone (a) and 5,6-DMI (b) by DFT method**

Supplement: Figure S3 [file mmc3.docx]

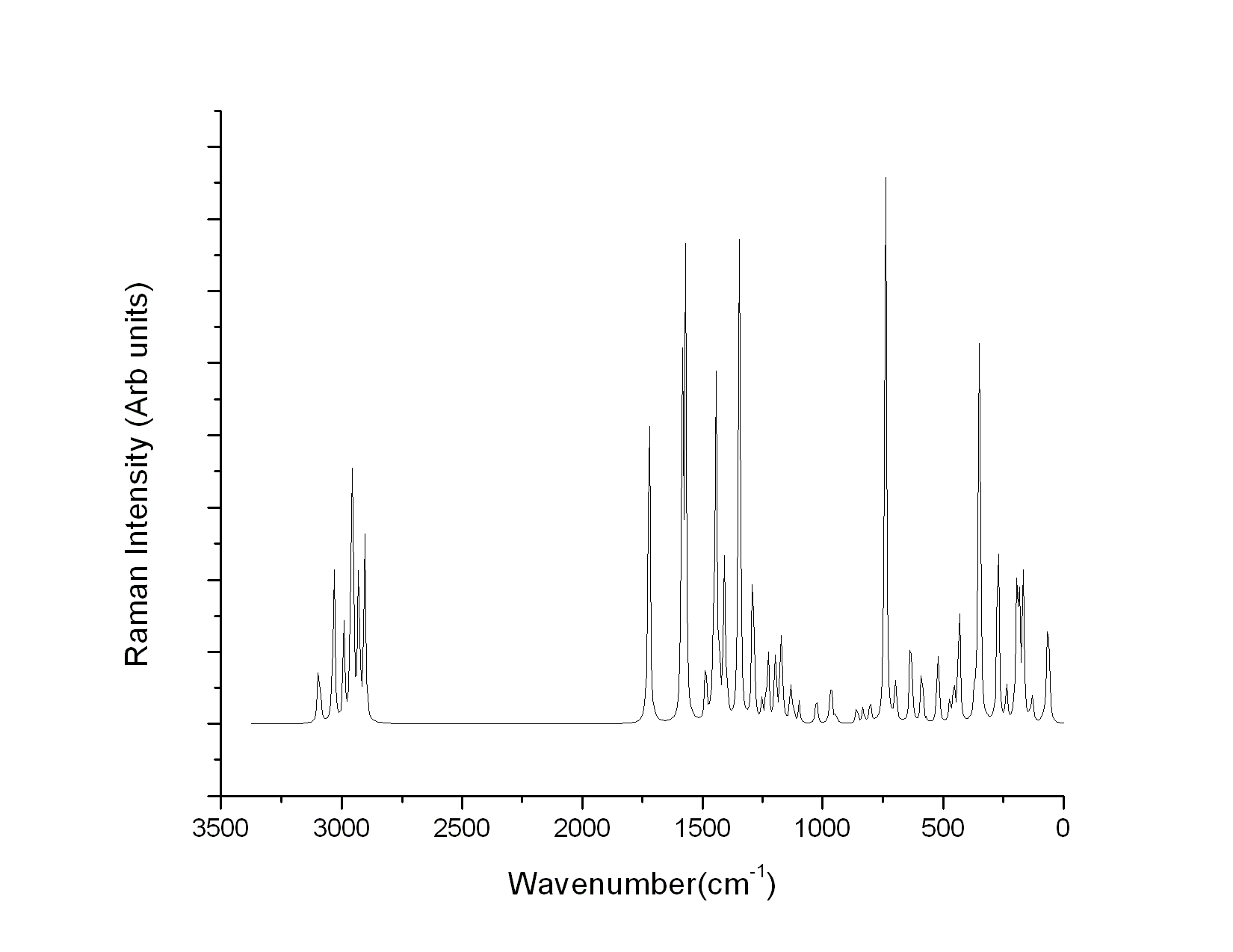


**(a)**


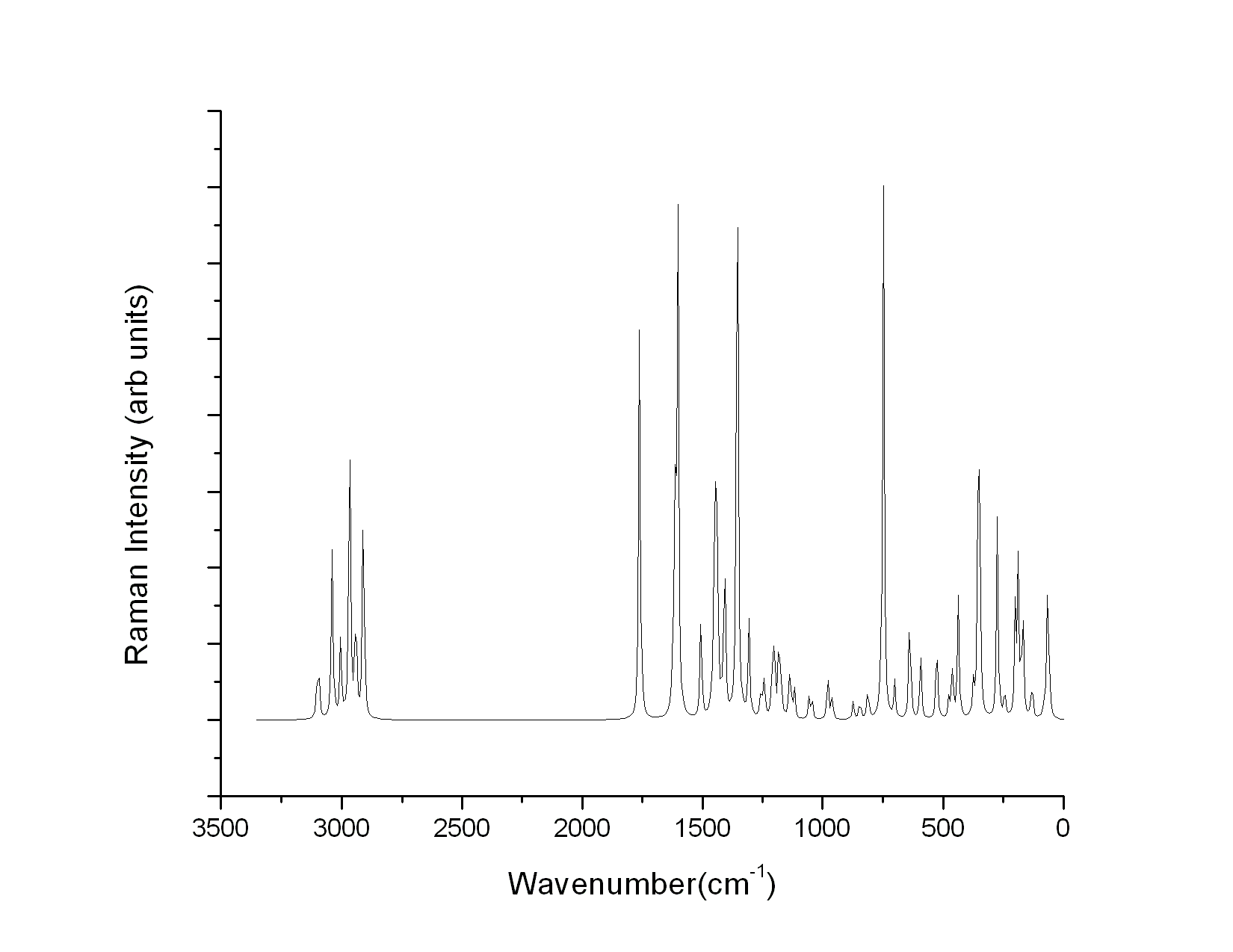


**(b)**

**Figure S5. Comparison Theoretical FT-Raman spectra by B3LYP/6-311G(d,p) (a) and CAM-B3LYP/6-311G(d,p) (b) by 5,6-DMI**

Supplement: Figure S5 [file mmc5.doc]
