# Supplementary material for: Conformational analysis, molecular structure, spectroscopic, NBO, reactivity descriptors, wavefunction and molecular docking investigations of 5,6-dimethoxy-1-indanone: A potential anti Alzheimer's agent
Source: Heliyon. 2022 Jan 23;8(1):e08821. doi: 10.1016/j.heliyon.2022.e08821 (PMC8808071; doi:10.1016/j.heliyon.2022.e08821)
Supplement: Figure S2 [file mmc2.docx]

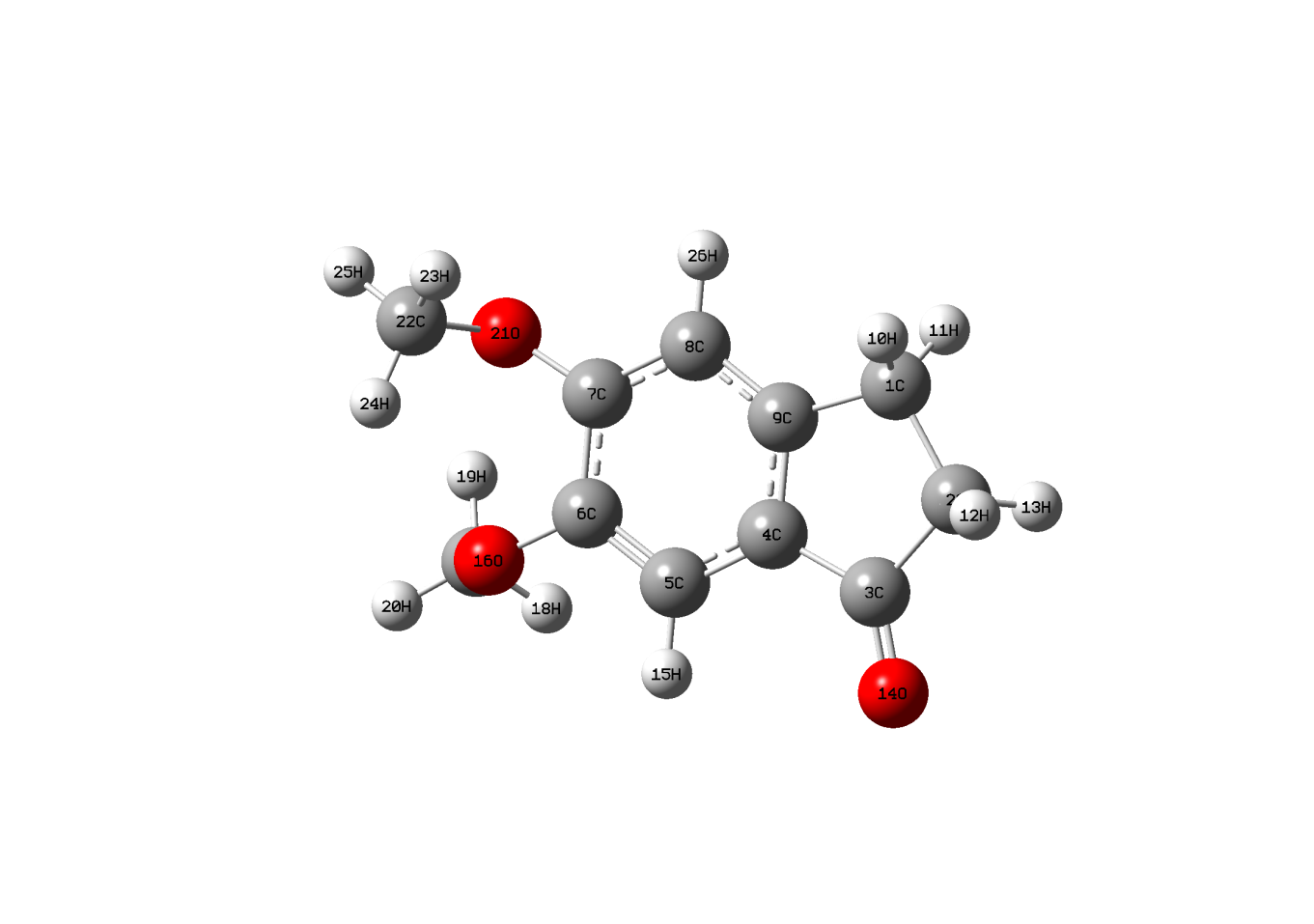

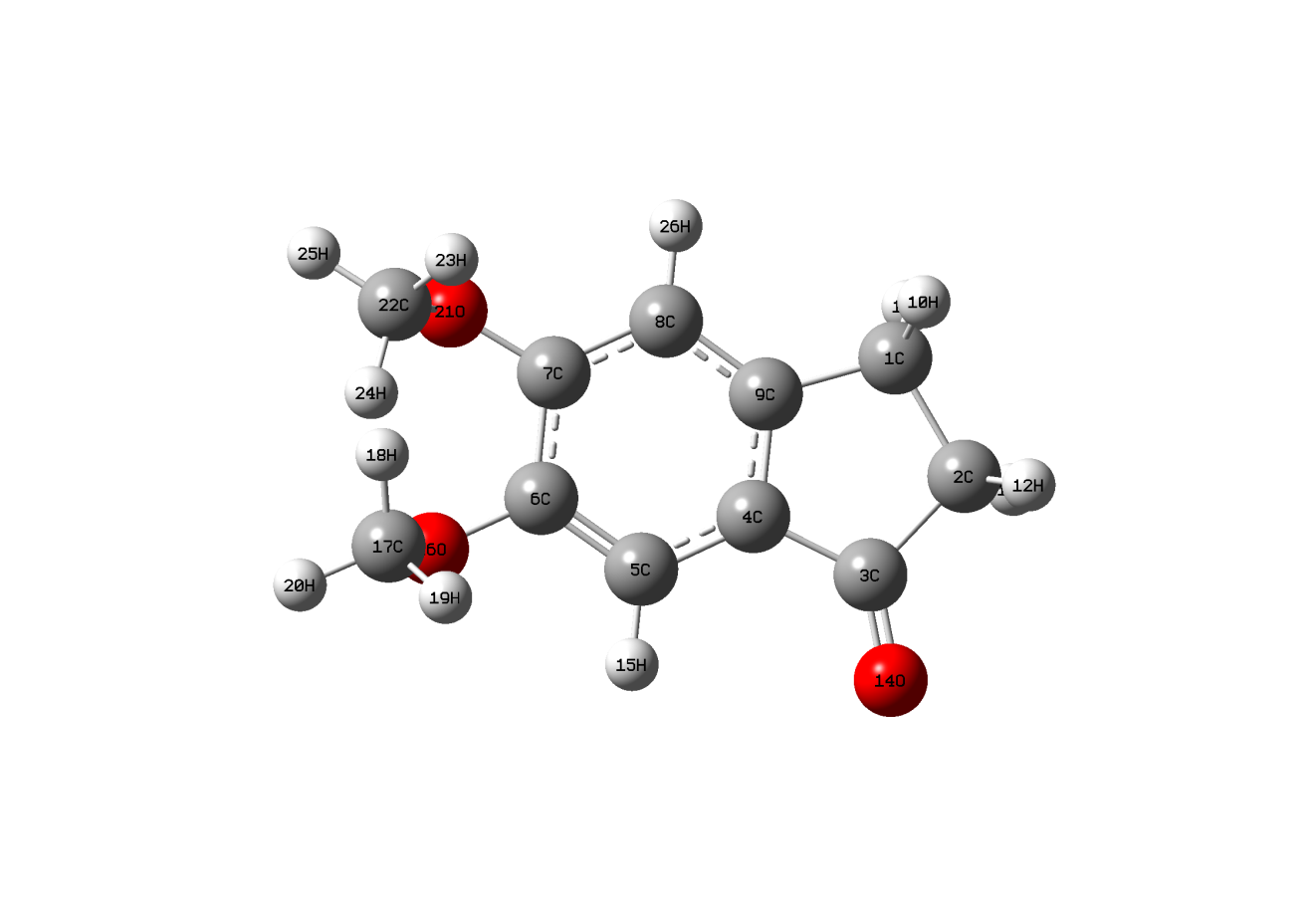

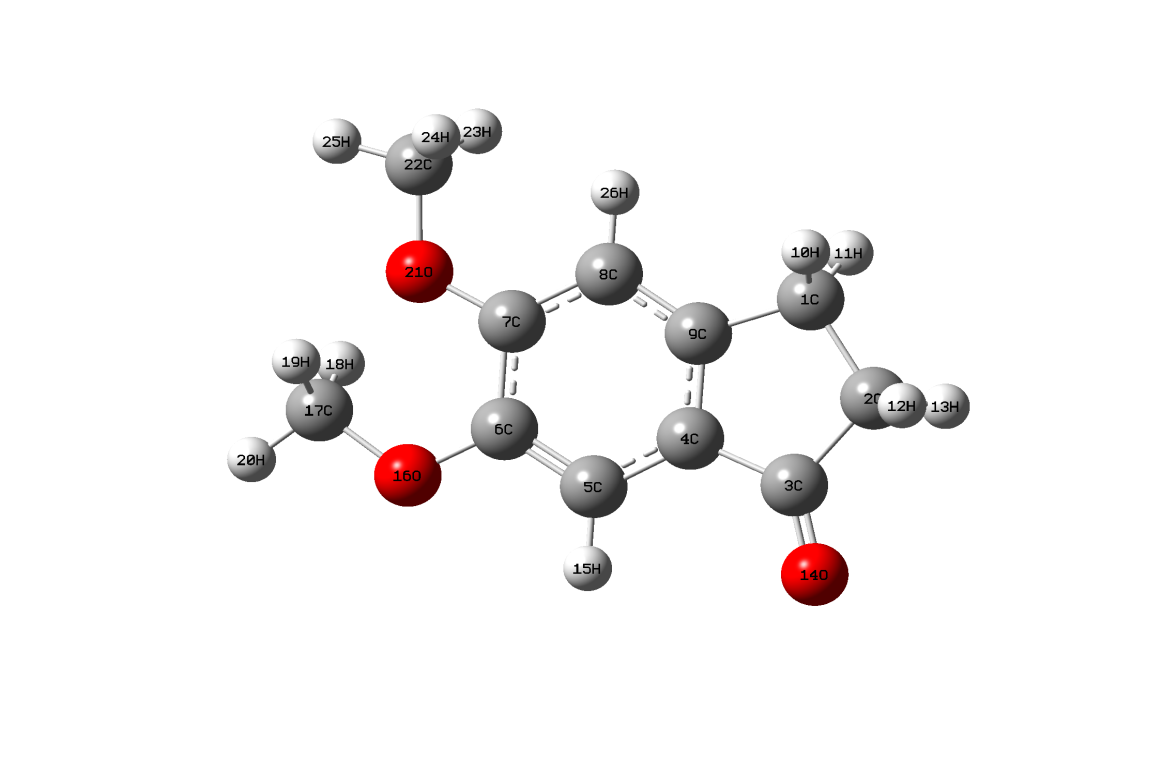

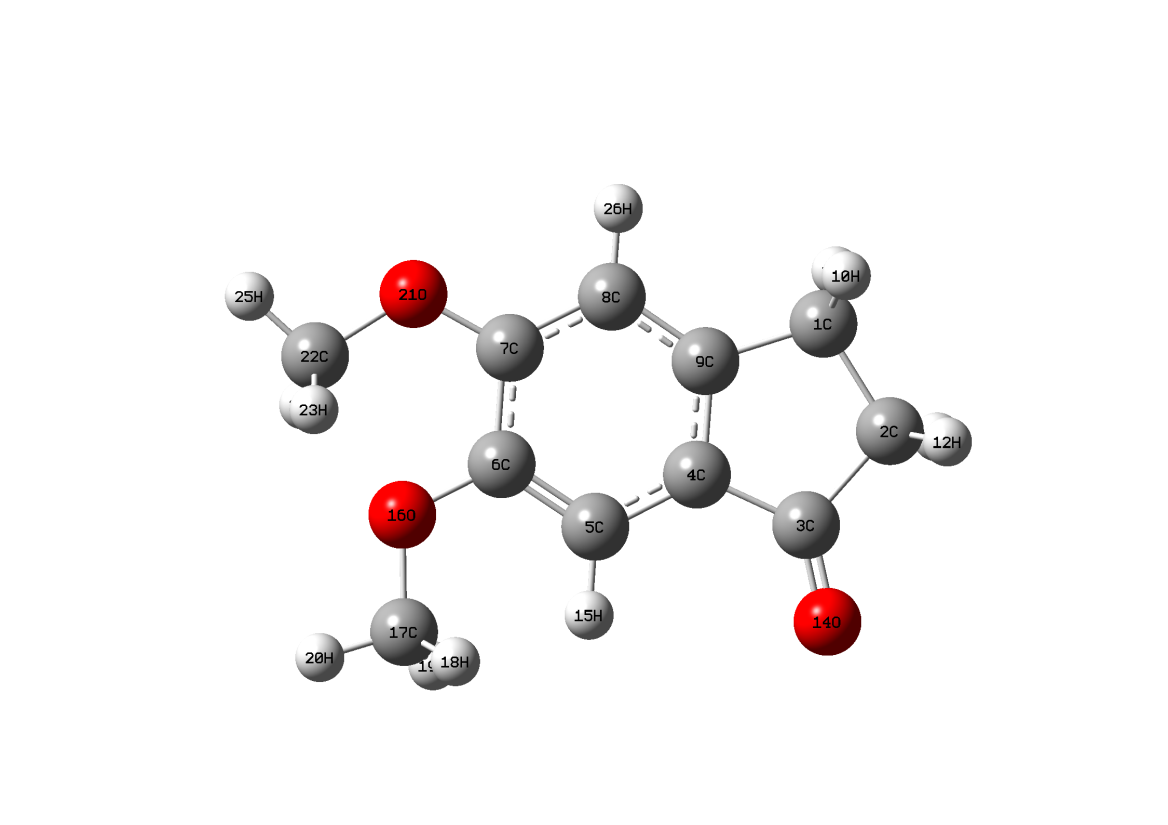

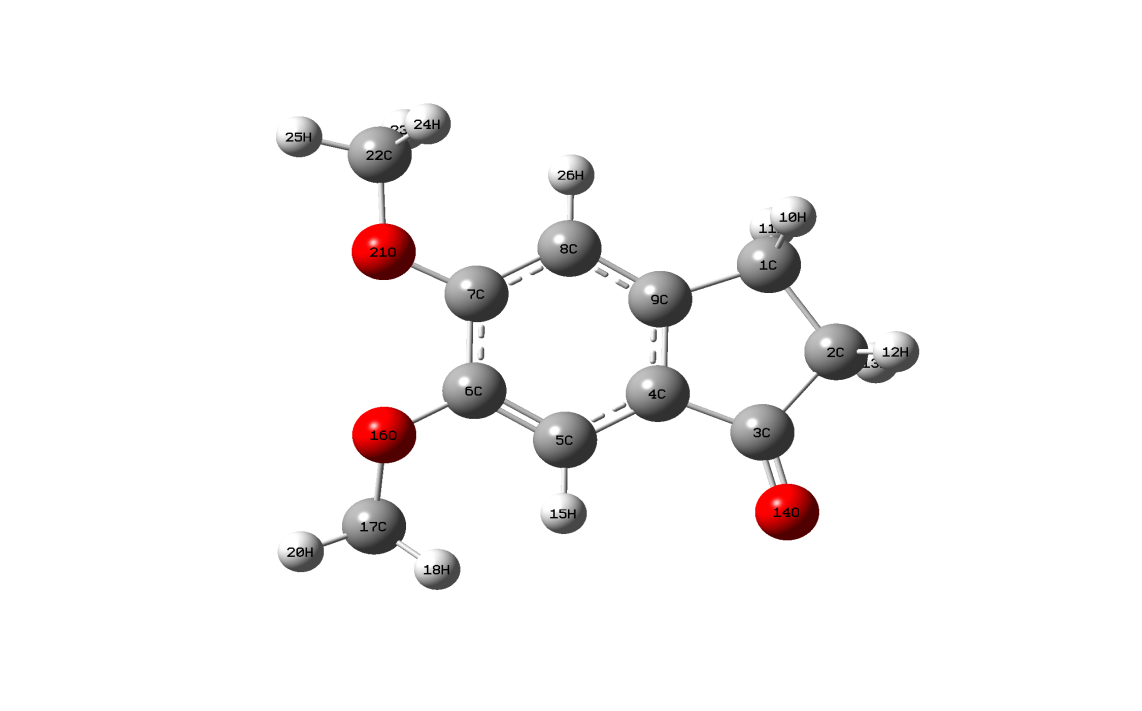


**Figure S2. Different Possible Conformer of 5,6-DMImolecule computed by CAM-B3LYP/6-311G(d,p) method**

**Most stable Conformer**

**Conformer 5**E= -408738.372092Kcalmol^-1^

**Conformer 4**E= -408739.548808Kcalmol^-1^

**Conformer 3**E=- -408739.750476Kcalmol^-1^

**Conformer 2**E= -408739.548758Kcalmol^-1^

**Conformer 1**E= -408741.166142Kcalmol^-1^

C1

C1

C1
