# Supplementary material for: Conformational analysis, molecular structure, spectroscopic, NBO, reactivity descriptors, wavefunction and molecular docking investigations of 5,6-dimethoxy-1-indanone: A potential anti Alzheimer's agent
Source: Heliyon. 2022 Jan 23;8(1):e08821. doi: 10.1016/j.heliyon.2022.e08821 (PMC8808071; doi:10.1016/j.heliyon.2022.e08821)
Supplement: Figure S4 [file mmc4.doc]

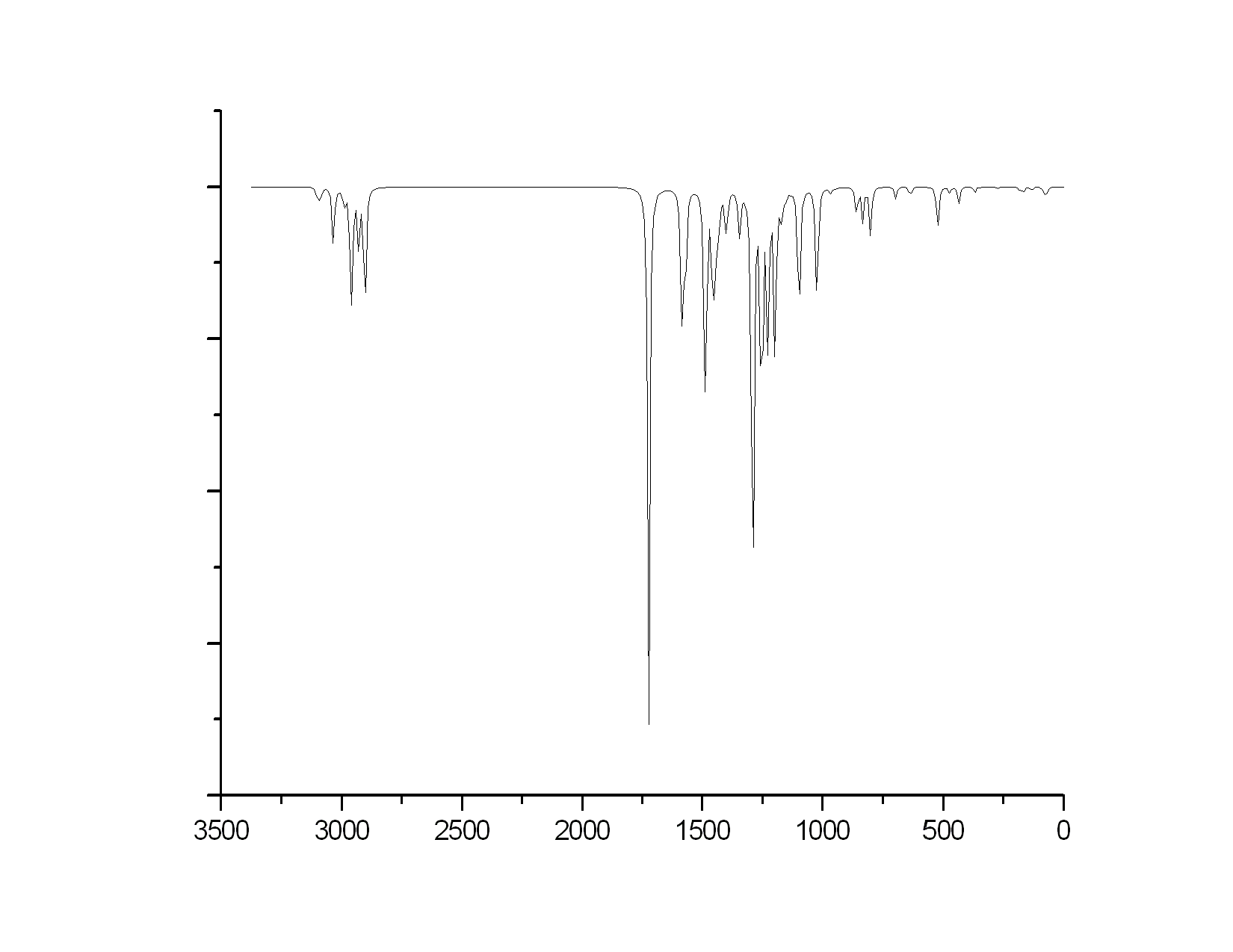


**(a)**

IR Intensity (Km/mol)


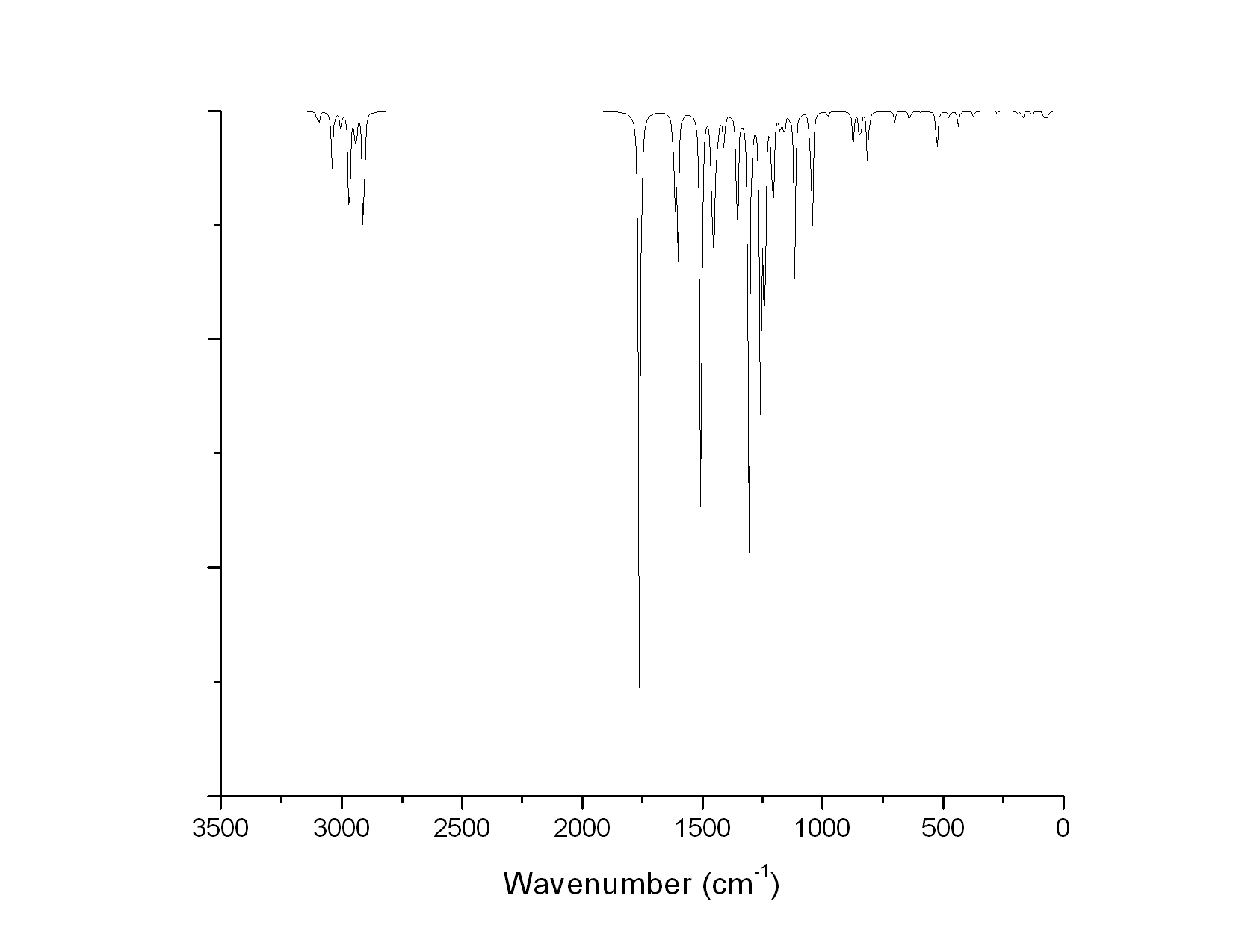


**(b)**

IR Intensity (Km/mol)

**Figure S4. Comparison Theoretical FT-IR spectra by B3LYP/6-311G(d,p) (a) and CAM-B3LYP/6-311G(d,p) (b) spectra of 5,6-DMI**
