# Supplementary material for: Conformational analysis, molecular structure, spectroscopic, NBO, reactivity descriptors, wavefunction and molecular docking investigations of 5,6-dimethoxy-1-indanone: A potential anti Alzheimer's agent
Source: Heliyon. 2022 Jan 23;8(1):e08821. doi: 10.1016/j.heliyon.2022.e08821 (PMC8808071; doi:10.1016/j.heliyon.2022.e08821)
Supplement: Figure S7 [file mmc7.docx]

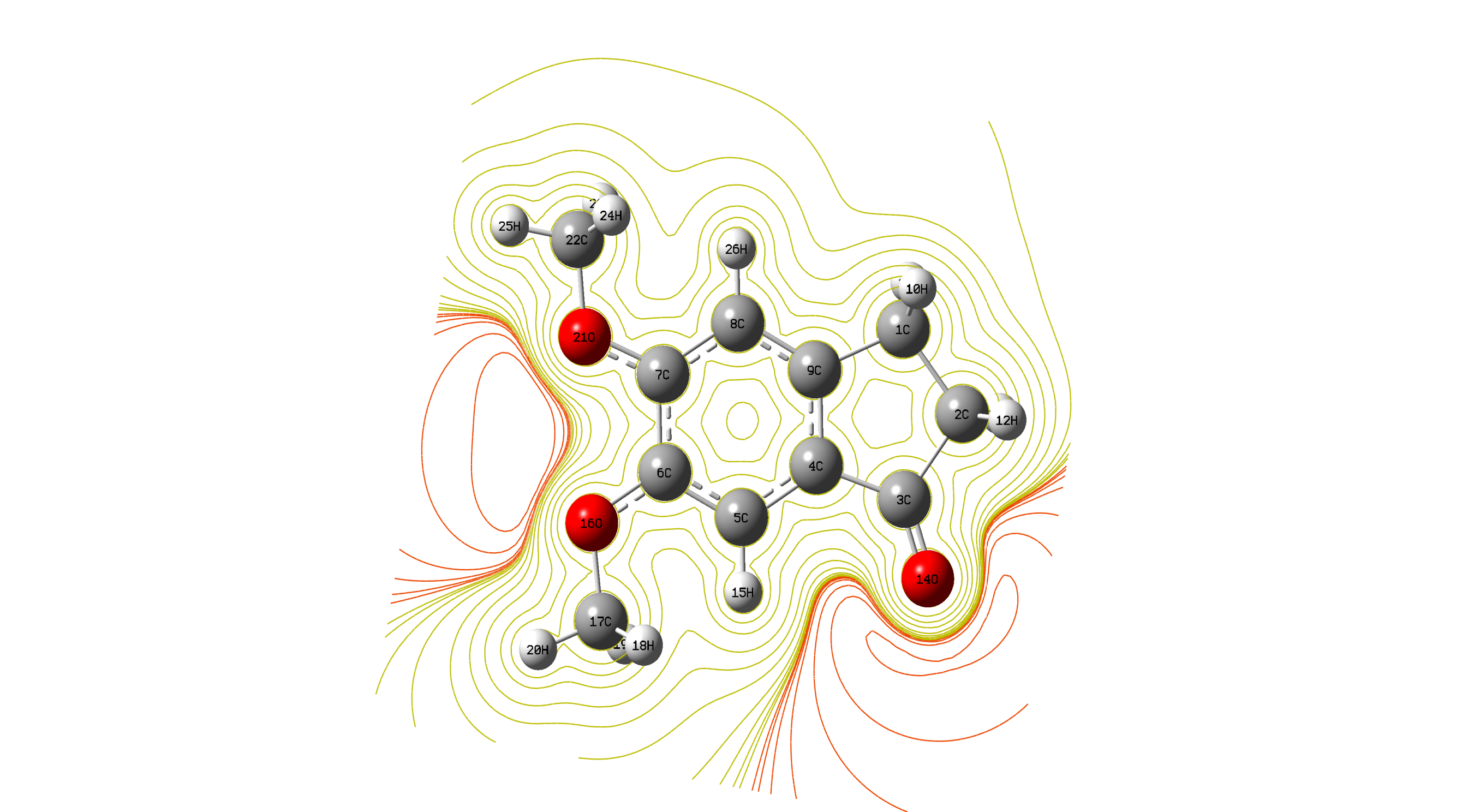

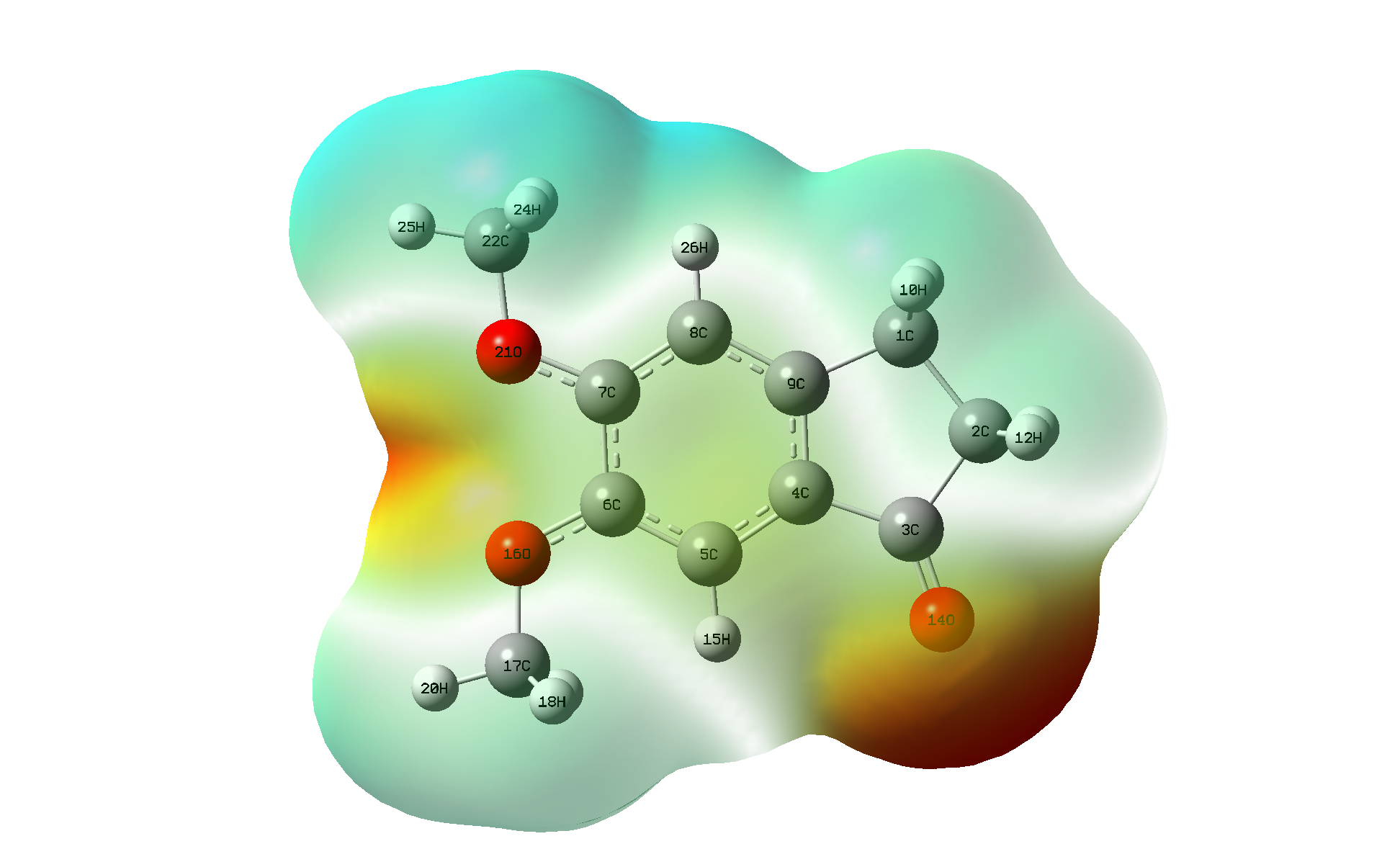

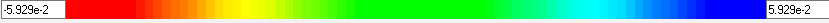


**Figure S7. Total Molecular Electrostatic Potential (MEP) (a) and Electrostatic Potential Contour diagram (b)
of 5,6-DMI**

**(b)**

**(a)**
