# Supplementary material for: Conformational analysis, molecular structure, spectroscopic, NBO, reactivity descriptors, wavefunction and molecular docking investigations of 5,6-dimethoxy-1-indanone: A potential anti Alzheimer's agent
Source: Heliyon. 2022 Jan 23;8(1):e08821. doi: 10.1016/j.heliyon.2022.e08821 (PMC8808071; doi:10.1016/j.heliyon.2022.e08821)
Supplement: Figure S8 [file mmc8.docx]

**(b)**

**(a)**

**(a)**

**Figure S8. Correlation graph of computed Heat Capacity, Entropy and Enthalpy for the 5,6-DMI by B3LYP/6-311G(d,p) (a) and CAM-B3LYP/6-311G(d,p) (b) method.**
