# Supplementary material for: Conformational analysis, molecular structure, spectroscopic, NBO, reactivity descriptors, wavefunction and molecular docking investigations of 5,6-dimethoxy-1-indanone: A potential anti Alzheimer's agent
Source: Heliyon. 2022 Jan 23;8(1):e08821. doi: 10.1016/j.heliyon.2022.e08821 (PMC8808071; doi:10.1016/j.heliyon.2022.e08821)
Supplement: Table S1 [file mmc9.doc]

**Table S1. Total and relative energies of different conformations of 5,6-DMI calculated at by CAM-B3LYP/6-311G(d,p) level of theory**

| **S.No** | **Conformer** | **Energy (kcalmol^-1^)** | |
| --- | --- | --- | --- |
|  |  | **CAM-B3LYP/6-311G(d,p)** | **Energy Difference (relative energy)** |
| 1 | C1 | -408741.166142^a^ | 0.0000 |
| 2 | C2 | -408739.548758 | 1.61738 |
| 3 | C3 | -408739.750476 | 1.41567 |
| 4 | C4 | -408739.548808 | 1.61733 |
| 5 | C5 | -408738.372092 | 2.79405 |

**^a^ Global minimum energy**
