# Supplementary material for: Conformational analysis, molecular structure, spectroscopic, NBO, reactivity descriptors, wavefunction and molecular docking investigations of 5,6-dimethoxy-1-indanone: A potential anti Alzheimer's agent
Source: Heliyon. 2022 Jan 23;8(1):e08821. doi: 10.1016/j.heliyon.2022.e08821 (PMC8808071; doi:10.1016/j.heliyon.2022.e08821)
Supplement: Table S2 [file mmc10.docx]

**Table S2 NBO charge analysis of 5,6-DMI and Indanone by DFT method.**

| **5,6-DMI** | | | **Indanone** | | |
| --- | --- | --- | --- | --- | --- |
| **Charge(e)** | | | **Charge(e)** | | |
| **Atom Number** | **B3LYP/6-311G(d,p)** | **CAM-B3LYP/6-311G(d,p)** | **Atom Number** | **B3LYP/6-311G(d,p)** | **CAM-B3LYP/6-311G(d,p)** |
| C1 | -0.3961 | -0.4029 | C1 | -0.3779 | -0.3856 |
| C2 | -0.4672 | -0.4770 | C2 | -0.4535 | -0.4621 |
| C3 | 0.5745 | 0.5918 | C3 | 0.58337 | 0.59829 |
| C4 | -0.1687 | -0.1765 | C4 | -0.1471 | -0.1532 |
| C5 | -0.2506 | -0.2532 | C5 | -0.1597 | -0.1619 |
| C6 | 0.2964 | 0.2982 | C6 | -0.2003 | -0.2057 |
| C7 | 0.3368 | 0.3427 | C7 | -0.1536 | -0.1565 |
| C8 | -0.3061 | -0.3131 | C8 | -0.2144 | -0.2201 |
| C9 | 0.0278 | 0.0307 | C9 | 0.01699 | 0.01734 |
| H10 | 0.2071 | 0.2107 | H10 | 0.2045 | 0.20794 |
| H11 | 0.2071 | 0.2107 | H11 | 0.20247 | 0.20646 |
| H12 | 0.2212 | 0.2260 | H12 | 0.2151 | 0.21944 |
| H13 | 0.2213 | 0.2260 | H13 | 0.21749 | 0.22126 |
| O14 | -0.5667 | -0.5716 | O14 | -0.5545 | -0.562 |
| H15 | 0.2249 | 0.2292 | H15 | 0.21499 | 0.21925 |
| O16 | -0.5125 | -0.5189 | H16 | 0.20529 | 0.20919 |
| C17 | -0.1967 | -0.2048 | H17 | 0.19904 | 0.20271 |
| H18 | 0.1659 | 0.1682 | H18 | 0.2016 | 0.20499 |
| H19 | 0.1659 | 0.1682 |  |  |  |
| H20 | 0.1855 | 0.1889 |  |  |  |
| O21 | -0.5029 | -0.5103 |  |  |  |
| C22 | -0.1975 | -0.2056 |  |  |  |
| H23 | 0.1657 | 0.1679 |  |  |  |
| H24 | 0.1657 | 0.1679 |  |  |  |
| H25 | 0.1882 | 0.1916 |  |  |  |
| H26 | 0.2112 | 0.2151 |  |  |  |
