# Supplementary material for: Conformational analysis, molecular structure, spectroscopic, NBO, reactivity descriptors, wavefunction and molecular docking investigations of 5,6-dimethoxy-1-indanone: A potential anti Alzheimer's agent
Source: Heliyon. 2022 Jan 23;8(1):e08821. doi: 10.1016/j.heliyon.2022.e08821 (PMC8808071; doi:10.1016/j.heliyon.2022.e08821)
Supplement: Table S3 [file mmc11.doc]

| **Donor (i)** | **Type** | **ED/e** | **Acceptor (j)** | **Type** | **ED/e** | **E^(2)a^(KJmol^-1^)** | **E(j)-E(i)^b^(a.u)** | **F(i,j)^c^ (a.u)** |
| --- | --- | --- | --- | --- | --- | --- | --- | --- |
| C1-C2 | σ | 1.98114 | C3-O14 | σ^*^ | 0.00740 | 3.76 | 1.37 | 0.064 |
|  |  |  | C8-C9 | σ^*^ | 0.02502 | 5.13 | 1.32 | 0.074 |
| C1-C9 | σ | 1.97370 | C4-C5 | σ^*^ | 0.02607 | 4.95 | 1.34 | 0.073 |
|  |  |  | C4-C9 | σ^*^ | 0.02928 | 2.88 | 1.37 | 0.056 |
|  |  |  | C7-C8 | σ^*^ | 0.02514 | 2.34 | 1.32 | 0.050 |
|  |  |  | C8-C9 | σ^*^ | 0.02502 | 3.02 | 1.34 | 0.057 |
| C2-C3 | σ | 1.98204 | C4-C5 | σ^*^ | 0.02607 | 4.55 | 1.33 | 0.069 |
| C3-C4 | σ | 1.97517 | C4-C5 | σ^*^ | 0.02607 | 3.86 | 1.36 | 0.065 |
|  |  |  | C4-C9 | σ^*^ | 0.02928 | 3.87 | 1.39 | 0.066 |
|  |  |  | C8-C9 | σ^*^ | 0.02502 | 4.71 | 1.37 | 0.072 |
| C3-O14 | σ | 1.99554 | C3-C4 | σ^*^ | 0.06988 | 2.11 | 1.70 | 0.054 |
|  | π | 1.98087 | C4-C9 | π^*^ | 0.36092 | 4.66 | 0.52 | 0.048 |
| C4-C5 | σ | 1.96682 | C3-C4 | σ^*^ | 0.06988 | 3.62 | 1.30 | 0.062 |
|  |  |  | C4-C9 | σ^*^ | 0.02928 | 6.87 | 1.44 | 0.089 |
|  |  |  | C6-O16 | σ^*^ | 0.02717 | 5.53 | 1.20 | 0.073 |
| C4-C9 | σ | 1.96530 | C4-C5 | σ^*^ | 0.02607 | 6.54 | 1.41 | 0.086 |
|  | π | 1.67532 | C3-O14 | π^*^ | 0.15397 | 2.99 | 1.46 | 0.059 |
|  |  |  | C5-C6 | π^*^ | 0.31856 | 26.43 | 0.37 | 0.088 |
|  |  |  | C7-C8 | π^*^ | 0.35068 | 22.92 | 0.36 | 0.081 |
| C5-C6 | σ | 1.97724 | C3-C4 | σ^*^ | 0.06988 | 3.93 | 1.33 | 0.065 |
|  |  |  | C4-C5 | σ^*^ | 0.02607 | 4.88 | 1.44 | 0.075 |
|  |  |  | C6-C7 | σ^*^ | 0.03607 | 5.03 | 1.37 | 0.074 |
|  | π | 1.72694 | C4-C9 | π^*^ | 0.36092 | 24.85 | 0.40 | 0.091 |
|  |  |  | C7-C8 | π^*^ | 0.35068 | 25.95 | 0.38 | 0.090 |
| C7-C8 | σ | 1.97638 | C1-C9 | σ^*^ | 0.02470 | 4.77 | 1.28 | 0.070 |
|  |  |  | C6-C7 | σ^*^ | 0.03607 | 4.81 | 1.37 | 0.073 |
|  | π | 1.71145 | C4-C9 | π^*^ | 0.36092 | 31.48 | 0.40 | 0.103 |
|  |  |  | C5-C6 | π^*^ | 0.31856 | 20.71 | 0.39 | 0.081 |
| O14 | LP(2) | 1.99975 | C2-C3 | σ^*^ | 0.06726 | 2.18 | 1.17 | 0.046 |
|  |  |  | C2-C3 | σ^*^ | 0.06726 | 26.95 | 0.76 | 0.129 |
|  |  |  | C3-C4 | σ^*^ | 0.06988 | 22.81 | 0.85 | 0.126 |
| O16 | LP(2) | 1.84640 | C5-C6 | σ^*^ | 0.02417 | 7.72 | 1.26 | 0.088 |
|  |  |  | C5-C6 | π^*^ | 0.31856 | 37.68 | 0.45 | 0.121 |
| O21 | LP(2) | 1.83256 | C7-C8 | σ^*^ | 0.02514 | 7.78 | 1.24 | 0.088 |
|  |  |  | C7-C8 | π^*^ | 0.35068 | 40.85 | 0.44 | 0.126 |

**Table S3 . Second Order Perturbation Theory Analysis of Fock Matrix in NBO Basis for 5,6-DMI by CAM-B3LYP/6-311G(d,p) method.**

^a^E^(2)^ means energy of hyper conjugative interaction (stabilization energy)

^b^Energy difference between donor and acceptor i and j NBO orbitals.

^c^F(i,j) is the Fork matrix element between i and j NBO orbitals.
