# Supplementary material for: Conformational analysis, molecular structure, spectroscopic, NBO, reactivity descriptors, wavefunction and molecular docking investigations of 5,6-dimethoxy-1-indanone: A potential anti Alzheimer's agent
Source: Heliyon. 2022 Jan 23;8(1):e08821. doi: 10.1016/j.heliyon.2022.e08821 (PMC8808071; doi:10.1016/j.heliyon.2022.e08821)
Supplement: Table S4 [file mmc12.docx]

**Table S4: HOMO – LUMO energies and calculated global reactive parameters of 5,6DMI molecule computed by B3LYP/6-311G(d,p) method in Gas and Solvent Phase**

| **Parameters** | **B3LYP/6-311G(d,p)** | | **CAM-B3LYP/6-311G(d,p)** | |
| --- | --- | --- | --- | --- |
|  | **Gas Phase** | **Ethanol Phase** | **Gas Phase** | **Ethanol Phase** |
| E _HOMO_ (eV) | -5.92 | -6.05 | -7.27 | -7.41 |
| E _LUMO_(eV) | -1.27 | -1.42 | 0.02 | -0.18 |
| E_HOMO_-E_LUMO_(eV) | 4.65 | 4.63 | 7.25 | 7.23 |
| E _HOMO_-1(eV) | -6.52 | -6.86 | -8.25 | -8.42 |
| E _LUMO_+1(eV) | -0.2 | -0.23 | 1.12 | 1.05 |
| E_HOMO-1_ –E_LUMO+1_(eV) | 6.50 | 6.63 | 7.13 | 7.37 |
| Eectronegativity($\chi$) | 2.33 | 2.31 | 3.64 | 3.79 |
| Chemical hardness ($\eta$) | 2.32 | 2.31 | 3.62 | 3.61 |
| Softness(S) | 0.43 | 0.43 | 0.27 | 0.27 |
| Chemical Potential ($\mu$) | -2.33 | -2.31 | -3.62 | -3.61 |
| Electrophilicity index ($\varpi$) | 1.170 | 1.155 | 1.810 | 1.805 |
