# Supplementary material for: Conformational analysis, molecular structure, spectroscopic, NBO, reactivity descriptors, wavefunction and molecular docking investigations of 5,6-dimethoxy-1-indanone: A potential anti Alzheimer's agent
Source: Heliyon. 2022 Jan 23;8(1):e08821. doi: 10.1016/j.heliyon.2022.e08821 (PMC8808071; doi:10.1016/j.heliyon.2022.e08821)
Supplement: Table S5 [file mmc13.docx]

**Table S5. Frequently considered drug likeness parameters calculated**

| **Descriptor** | **Value** |
| --- | --- |
| Number of Hydrogen bond Acceptor | 3 |
| Number of Hydrogen bond donar | 0 |
| Alog P | 2.21 |
| Molecular weight (g/mol) | 192.21 |
| Molar refractivity | 52.47 |
| Number of rotatable bonds | 2 |
| Topological polar surface area (Ǻ^2^) | 35.53 |
| Number of heavy atoms | 14 |
