# Supplementary material for: Conformational analysis, molecular structure, spectroscopic, NBO, reactivity descriptors, wavefunction and molecular docking investigations of 5,6-dimethoxy-1-indanone: A potential anti Alzheimer's agent
Source: Heliyon. 2022 Jan 23;8(1):e08821. doi: 10.1016/j.heliyon.2022.e08821 (PMC8808071; doi:10.1016/j.heliyon.2022.e08821)
Supplement: Table S6 _spl_ S7 [file mmc14.docx]

| **Temp** | **C(Calmol^-1^K^-1^)** | **S(Cal mol^-1^K^-1^)** | **H (k Cal/molK^-1^)** |
| --- | --- | --- | --- |
| 100 | 20.512 | 74.650 | 132.825 |
| 200 | 34.501 | 94.601 | 135.584 |
| 298.15 | 48.464 | 111.744 | 139.65 |
| 400 | 62.808 | 128.608 | 145.326 |
| 500 | 75.229 | 144.439 | 152.245 |
| 600 | 85.582 | 159.462 | 160.302 |
| 700 | 94.112 | 173.623 | 169.301 |

| **Temp** | **C(Calmol^-1^K^-1^)** | **S(Cal mol^-1^K^-1^)** | **H (k Cal/molK^-1^)** |
| --- | --- | --- | --- |
| 100 | 20.221 | 74.464 | 134.548 |
| 200 | 34.009 | 94.155 | 137.269 |
| 298.15 | 47.742 | 111.063 | 141.275 |
| 400 | 61.944 | 127.685 | 146.869 |
| 500 | 74.34 | 143.319 | 153.7 |
| 600 | 84.727 | 158.183 | 161.669 |
| 700 | 93.312 | 172.216 | 170.585 |

**Table S6 Temperature dependent properties of 5,6-DMI at different temperature
 calculated at B3LYP/6-311G(d,p) method.**

**Table S7 Temperature dependent properties of 5,6-DMI at different temperature
 calculated at CAM-B3LYP/6-311G(d,p) method.**
